# Development and content validity of the evaluation of multidimensional functioning and risks in aging scale

**DOI:** 10.7717/peerj.20108

**Published:** 2025-12-09

**Authors:** José Fierro-Marrero, Álvaro Reina-Varona, Joaquín Pardo-Montero, Alba Paris-Alemany, Roy La Touche

**Affiliations:** 1 Departamento de Fisioterapia, Centro Superior de Estudios Universitarios La Salle, Universidad Autónoma de Madrid, Aravaca, Madrid, Spain; 2 Motion in Brains Research Group, Centro Superior de Estudios Universitarios La Salle, Universidad Autónoma de Madrid, Aravaca, Madrid, Spain; 3 Doctoral School, Universidad Autónoma de Madrid, Madrid, Spain; 4 Hospital La Paz Institute for Health Research–IdiPAZ (La Paz University Hospital–Universidad Autónoma de Madrid–Getafe Universitary Hospital—Universidad Europea de Madrid), Madrid, Spain; 5 Department of Radiology, Rehabilitation and Physiotherapy. Faculty of Nursery, Physiotherapy and Podiatry, Complutense University of Madrid, Madrid, Spain; 6 Instituto de Dolor Craneofacial y Neuromusculoesquelético (INDCRAN), Madrid, Spain

**Keywords:** Scale development, Validity, Content validity, Functioning, Frailty, Vulnerability, Geriatrics, Older adults, Aging

## Abstract

**Introduction:**

Rising life expectancy has led to an increased prevalence of age-related conditions such as pain, dementia, and falls. To address these challenges, healthcare systems require efficient tools to identify which health domains are preserved or impaired in older adults. Existing frailty instruments present both conceptual and operational limitations. Therefore, there is a need to shift toward domain-specific evaluations of functioning and potential risks, aligned with established protocols such as the Comprehensive Geriatric Assessment and framed within the International Classification of Functioning, Disability and Health (ICF). This study aimed to develop the Evaluation of Multidimensional Functioning and Risks in Aging (EMFRA) scale, encompassing four assessment domains: physical function, cognitive function, emotional status, and social situation.

**Methods:**

The EMFRA scale was developed by identifying potential items through a comprehensive literature review and expert input. The first preliminary version was validated by a panel of 15 experts, who assessed the scale’s clarity, coherence, and relevance using a 5-point Likert scale. Cognitive interviews were then conducted with 10 clinicians and 10 older adults to evaluate the comprehensibility and practical applicability of the second preliminary version.

**Results:**

Following the literature review, 24 items were grouped into four domains (six items per domain), each supported by evidence linking them to health-related adverse outcomes. Expert evaluation showed substantial agreement on comprehension, coherence, and relevance (Aiken’s V >0.7) for all but two items—language and fear—which were excluded. Cognitive interviews led to the exclusion of one additional item (sedentarism) and further refinement of the remaining items. These changes were incorporated into the final version of EMFRA, enhancing its usability and comprehensiveness.

**Conclusion:**

EMFRA provides a multidimensional framework for assessing functioning and risks in older adults, capturing physical, cognitive, emotional, and social factors. The inclusion of end-user feedback ensured the scale’s practical relevance. However, the current version of EMFRA should not yet be used in clinical practice, as further psychometric validation is required to confirm its utility.

## Introduction

Life expectancy has increased together with life span, leading to an increase in older populations worldwide (Aburto et al., 2020; Ho & Hendi, 2018). This demographic trend brings with it a growing prevalence of conditions such as pain (Zimmer et al., 2022), dementia (GBD 2019 Dementia Forecasting Collaborators, 2022), falls (Ghasemi et al., 2025), which frequently peak in advanced age. Consequently, healthcare systems face the challenge of maintaining quality of life, autonomy and well-being in the older population.

To achieve this, accurate clinical assessment is essential for identifying individual needs, anticipating deterioration, and tailoring appropriate interventions. Evaluation methods grounded in valid and operational diagnostic frameworks are crucial for this purpose. One of the most recognized models in geriatric medicine is the Comprehensive Geriatric Assessment (CGA), which integrates multiple dimensions of health. CGA includes clinical examination (medical history, diseases, medication review), physical function and activity assessment, psychological evaluation (both emotional and cognitive), and social and environmental appraisal (Brown et al., 1988). This multidimensional evaluation has demonstrated effectiveness in reducing short-term mortality (6–12 months) (Stuck et al., 1993).

The domains addressed in CGA align with biopsychosocial model (Engel, 1977), and so with the evaluation of functioning described by the International Classification of Functioning, Disability and Health (ICF) (World Health Organization, 2001). The ICF conceptualizes “functioning” as *“an umbrella term encompassing all body functions, activities and participation”*. Unlike traditional biomedical models focused solely on disease, the ICF emphasizes on the physiological functioning, overt human functions, the interaction of the individual with the environment, and achieve autonomy in daily life. The inclusion of functioning in health assessment serves as a paradigm to explain and analyze beyond the limits of disease within the biomedical model (Meland & Hjörleifsson, 2024). Empirical evidence indicates that the level of functioning provides valuable information to predict dependency independently of the presence of disease (Guralnik et al., 1994; Liao et al., 2022).

However, despite this shift toward the biopsychosocial model, geriatrics has been highly influenced by the concept of frailty, remaining dominant in recent decades. Initially defined by through the frailty phenotype (Fried et al., 2001), and the accumulation deficit model (Mitnitski, Mogilner & Rockwood, 2001), emerged to two different models of frailty. A recent review that conducted a content analysis of frailty instruments, revealed that they evaluate a wide spectrum health related deficits, including body structures, diseases, functions, participation, sociodemographic, or environmental factors among others (Fierro-Marrero et al., 2025). These evaluations also provide great value to identify which health domains are affected in older adults.

Nevertheless, the term frailty itself remains highly ambiguous (Apóstolo et al., 2017; Rockwood, Hogan & MacKnight, 2000; Vass & Hendriksen, 2016) and inconsistently defined construct (Sobhani et al., 2021). Recent reviews have highlighted major methodological flaws, mainly related to a lack of content and structural validity (Faller et al., 2019), along with a highly heterogeneous items, and varied scoring methods (Fierro-Marrero et al., 2025). As a result, these tools frequently group individuals with disparate profiles under the same “frail” label, which complicates diagnosis, and hinders therapeutic decisions and the establishment of a prognosis. In addition, the total score does not clearly represent the clinical profile of the patient, and the responsiveness of these instruments lack clear interpretation (Fierro-Marrero et al., 2025).

Although frailty instruments offer a pragmatic value, serving as a screening tools to detect general health-related deficits, new approaches suggest focusing on evaluating specific health domains (Fierro-Marrero et al., 2025). This domain-based evaluation aligns more closely with the principles of the ICF and the CGA.

Given this context, there is a clear need for a new tool specifically focused on the evaluation of functioning in older adults. A rapid assessment tool based on functioning and potential risks could serve as a complementary assessment withing the CGA. This would help clinicians make timely decisions, either by guiding further assessments or by informing early therapeutic actions.

Given this context, there is a clear need for a new tool specifically focused on the evaluation of functioning in older adults. A rapid functioning-centered assessment could effectively complement the functional component of CGA. In addition, current frailty scales are limited by conceptual ambiguity, and their overall scores often lack clear responsiveness and clinical interpretation. In contrast, a domain-based approach makes it possible to identify which areas of functioning are preserved or impaired, allowing for more targeted and individualized care.

The aim of this study is to develop the Evaluation of Multidimensional Functioning and Risks in Aging scale (EMFRA) and to assess its content validity. EMFRA is intented as a rapid screening tool focused on functioning in older adults, structured around four core domains: physical functions, cognitive functions, emotional state, and social domain.

## Methods

### Evaluation of multidimensional functioning and risks in aging scale development

The development of the present scale was conducted under Consensus-based Standards for the Selection of Health Measurement Instruments (COSMIN) guidelines (De Vet et al., 2011). The present study was conducted under ethical considerations following the Helsinki declaration, and participants were included after signing the informed consent of participation. The study was approved by the Ethics Committee of the Hospital Clínico San Carlos, Madrid, Spain, with the identifier no23/398-E.

#### Search and synthesis of literature

A literature review was conducted in the PubMed and Google Scholar databases (in English and Spanish) with the following objectives: (1) identify previous tools for assessing multidimensional frailty; (2) identify another set of tools that assesses physical function, cognitive function, emotional status, and social status; (3) identify the measurement procedure for these variables; (4) analyze which of these assessments present a cross-sectional or longitudinal association with the risk of health-related adverse events (Fierro-Marrero et al., 2025).

#### Preliminary draft of evaluation of multidimensional functioning and risks in aging scale

Based on the information previously gathered, the selection of items for the first preliminary version of the EMFRA scale (EMFRA-P1) was conducted. This selection process was performed considering criteria of relevance and validity to ensure that the scale adequately reflected the constructs of interest in a functional evaluation.

The preliminary scale consisted of four assessment domains: physical function, cognitive function, emotional status, and social situation. Each of these domains included six items that enabled a multidimensional evaluation of the individual’s condition. The selection of these domains responded to the need to address the construct in an integrated manner, recognizing the interrelationship between various aspects of health and participant well-being.

We then defined the most appropriate assessment procedure for the preliminary scale. Three main modalities were considered: functional assessment through objective testing, self-administration by the participant, and hetero-administration by the evaluator. The choice of the type of administration was made based on the characteristics of the items and the ease of implementation in various evaluation contexts. Additionally, three response categories were established, which were based on the previously synthesized information, allowing for a simple and standardized measurement of participant responses.

This preliminary development was an important first step toward the construction of a robust functional assessment instrument, which enabled the identification of strengths and areas of need across various dimensions of human functioning, thereby contributing to a better understanding of the factors influencing functioning in the individuals evaluated.

### Content validation

#### Content validation by an expert panel

An expert panel of healthcare providers was formed, including 15 experts (Lynn, 1986). All experts were Spanish healthcare professionals, specialized in any field of knowledge. The inclusion criteria were deliberately broad to ensure a multidisciplinary perspective, reflecting the diverse professional backgrounds expected to use the scale (Davis, 1992; De Vet et al., 2011). Importantly, all panel members were independent from the development team, which enhances the objectivity of the validation process as recommended by COSMIN guidelines (De Vet et al., 2011). An online validation panel was conducted through the Cognitoforms platform for content validation of the EMFRA-P1.

The following information was extracted: (1) sociodemographic data, including age, sex, nationality, native language, country, territory or state of residence, academic level, profession, and current job; (2) years of clinical and research experience with older adults, other frail populations, managing assessment tools, and frailty assessment tools, due to their historical relevance in geriatrics; (3) the number of ongoing research projects related to frail older adults they were currently working on; and (4) the number of validation processes in which they had previously participated.

An initial presentation was provided, outlining the justification, objectives, and scope of the study, as well as informing participants about the study procedures through the informed consent process. The informed consent ensured that participants were fully aware of their role, the purpose of the research, and the voluntary nature of their involvement, following ethical guidelines for research with human subjects (Gupta, 2013).

The validation panel had the objective of systematically assessing several aspects of the preliminary version of the EMFRA-P1. Specifically, the panel focused on evaluating the following components:

**1. Comprehension**

• **Item/question understanding**: Evaluators were asked to determine whether each item or question was clearly phrased, easily understandable, and unambiguous. This step was crucial for ensuring that all respondents, regardless of their background, could consistently interpret the items in the intended manner. Effective comprehension minimizes measurement error and improves the reliability of self-reporting (Beatty & Willis, 2007).
• **Score/response option understanding**: This evaluation addressed whether participants could clearly understand the available response options for each question. It was necessary to determine whether the scoring was transparent and appropriately mapped to the construct being measured.

**2. Measurement procedure suitability**

• The appropriateness of the measurement procedures was assessed by considering whether the proposed approach for each item (*e.g.*, self-administration, hetero-administration, or functional testing) was feasible, valid, and suited to capturing the construct of interest. The evaluation procedure included a discussion on practicality in terms of the time required for administration, participant burden, and the potential for each procedure to yield accurate and reliable measurements of the domains being investigated.

**3. Coherence**

• **Item/question coherence regarding multidimensional functioning**: Each item was scrutinized to determine whether it adequately captured elements of multidimensional functioning. Physical, cognitive, emotional, and social domains, each contribute to an individual’s overall resilience or vulnerability (Junius-Walker et al., 2018). The panel evaluated whether the items effectively represented these components and whether any domain required further elaboration.
• **Score/response option coherence**: The evaluators also assessed whether the scoring or response options were logically aligned with the respective item. For example, a question assessing physical function should include response options that meaningfully capture various levels of ability, or an item assessing task difficulty should be answered through difficulty category levels.

**4. Relevance**

• **Relevance of items for assessing multidimensional functioning**: Relevance was considered in terms of how well each item contributed to the overall goal of assessing functioning across physical, cognitive, emotional and social dimensions. Items needed to reflect critical indicators of functioning, ensuring they collectively offered a comprehensive assessment of the construct. This step was intended to maintain the content validity of the scale, ensuring the instrument measures what it purports to measure (Haynes, Richard & Kubany, 1995).

To systematically capture the feedback from the validation panel, a 5-category Likert scale of agreement was used. The categories were as follows:

• “Totalmente en desacuerdo” (strongly disagree)
• “En desacuerdo” (disagree)
• “Ni de acuerdo ni en desacuerdo” (neither agree nor disagree)
• “De acuerdo” (agree)
• “Totalmente de acuerdo” (strongly agree)

This scale provided a nuanced view of the degree to which panel members concurred with the clarity, coherence, and relevance of each item, response option, and measurement procedure.

In addition to the Likert scale, the panel members were asked to respond to open-ended questions designed to gather suggestions, comments, and recommendations for modifications. These open-ended questions were critical for capturing insights beyond the structured assessment, allowing experts to provide detailed feedback on potential improvements. This approach aligns with the iterative nature of instrument development, in which continuous refinement based on expert input leads to greater validity and reliability of the final tool (DeVellis, 2017).

The feedback from the panel provided a comprehensive basis for refining the EMFRA scale, ensuring that the final version would be both scientifically robust and practically applicable for assessing functioning in diverse populations. The iterative process of validation and feedback is an essential component of psychometric scale development, emphasizing both empirical rigor and responsiveness to stakeholder perspectives.

#### Analysis and modification procedures after the expert panel

The level of agreement across authors for each of the previous points was explored with Aikens’ V (23):

\begin{eqnarray*}Aike{n}^{{^{\prime}}}s~V= \frac{S}{[n \left( c-1 \right) ]} . \end{eqnarray*}

The number of ordinal categories is denoted “c”, the number of evaluators “n”. “S” refers to the sum of the scores of the total number of evaluators. To calculate “S”, the score for the category with the level of agreement selected “ci” is subtracted from the value of the category with the lowest level of agreement, “c_low_”.

\begin{eqnarray*}S=\sum _{n}({c}_{i}-{c}_{low}). \end{eqnarray*}

A Likert scale for Level of Agreement was categorized with the following scores:

• “Totalmente en desacuerdo” (strongly disagree) = 1 point (c_low_)
• “En desacuerdo” (disagree) = 2 points
• “Ni de acuerdo ni en desacuerdo” (neither agree nor disagree) = 3 points
• “De acuerdo” (agree) = 4 points
• “Totalmente de acuerdo” (strongly agree) = 5 points

A low level of agreement across authors was considered when *V* < 0.7. Items were excluded if at least one question presented a low level of agreement across authors. Only items with all questions presenting a *V* ≥ 0.7 were maintained.

Maintained items were modified based on the suitability of the suggestions retrieved by the authors.

The list of suggestions made by every author was reported, indicating which suggestions were considered suitable for conducting modifications. A second preliminary version of the scale was named EMFRA-P2, highlighting the modifications conducted, which is presented Supplementary Material. EMFRA-P2 is laid out for administration to clinicians and older adults in cognitive interviews.

#### Content validation by clinicians and older adults

The EMFRA-P2 was administered and validated by clinicians and healthy older adults, employing cognitive interviews (Estefania & Zalazar-Jaime, 2018). This process aimed to assess the comprehension and coherence of items for both clinicians and older adults, for their respective implication in administration and evaluation.

A total of 10 clinicians, and 10 healthy older adults were interviewed using a non-probabilistic sampling. This sample size aligns with established recommendations for cognitive interviewing for identifying major issues of interpretation (Beatty & Willis, 2007).

*Interview methodology for clinicians.* No specific selection criteria were applied to the participating clinicians, except that they were required to have a native or C2 level proficiency in Spanish from Spain. This criterion ensured that their comprehension and evaluation of the scale would not be hindered by language barriers, thereby allowing an accurate exploration of the scale’s comprehensibility and coherence regardless of their level of clinical expertise.

The following data were collected from the clinicians:

1. Sociodemographic information: This included age, sex, nationality, native language, country of residence, autonomous community of residence, academic level, profession, and current employment. Collecting these data allowed for the characterization of the clinician sample and an understanding of the potential influence of sociodemographic factors on their assessment of the scale.
2. Clinical experience: Information regarding their clinical experience was gathered, including years of clinical practice, years of experience working with older adults, years of experience with other populations identified as frail, and years of experience using assessment tests and tools. These data were crucial for understanding the clinicians’ familiarity and comfort with frailty-related concepts and tools, which could influence their ability to effectively evaluate the scale.

Before commencing the interview, the clinicians were provided with a detailed explanation of the administration procedure for the scale. This included specifying which items were functional tests, which required hetero-administration, and outlining the procedure for calculating the total score. By ensuring the clinicians fully understood the process prior to assessment, the validity of their feedback on the scale’s usability was optimized.

The interview then proceeded with the clinicians reading each item along with its associated scoring or response options. During this process, they were prompted to provide detailed feedback on any aspects that could be improved or modified to enhance the clarity, coherence, and applicability of the scale items. Their insights were particularly valuable, given their frontline experience in assessing frailty in clinical settings.

After reviewing all the items, the clinicians were asked to provide a general evaluation of the scale based on the following aspects:

• **Perceived difficulty in administering the scale**: The clinicians were asked to reflect on the ease or difficulty associated with administering the scale to the intended population.
• **Perceived difficulty in calculating the total score**: The complexity involved in scoring the scale was also assessed.

The clinicians provided their responses using a 5-point Likert scale of perceived difficulty:

• “Muy difícil” (very difficult)
• “Bastante difícil” (quite difficult)
• “Algo difícil” (somewhat difficult)
• “Poco difícil” (slightly difficult)
• “Nada difícil” (not difficult at all)

*Interview methodology for older adults.* The older adults were included based on the following criteria: (1) native or C2 level of Spanish from Spain; (2) age ≥65 years; (3) being cognitively healthy, considered when presenting ≥26 points on the Montreal Cognitive Assessment (MoCA) (Nasreddine et al., 2005; Sun et al., 2023). Subjects were recruited from La Salle Clinica Universitaria, Aravaca, Madrid, Spain.

Exclusion criteria included (1) any health condition posing risks when conducting physical tests or physical exercise (*e.g.*, infection, heart failure, pulmonary hypertension, dilated cardiomyopathy); (2) inability to stand without the assistance; (3) inability to walk independently even with assistive walking devices; (4) inability to transfer from sitting to standing even with the use of assistive walking devices; (5) inability to read or understand the investigators’ commands; or (6) inability to observe with clarity.

The healthy older adults were asked for sociodemographic data, including age, sex, height, weight, body mass index, completed educational level, years studied, marital status, and employment status.

At the start of the interview, the older adults were informed that they would be administered the scale. The administrator of the scale (a physiotherapist) was in charge of conducting the interview. The older adults were required to provide commentaries for modification. Additionally, the interviewer would annotate whether participants were able or not to respond to every item. After the older adults had responded to the question, they were asked to employ the previous 5-category Likert scale of difficulty to score two questions:

• Level of difficulty for comprehending the task/question
• Level of difficulty for comprehending response options.

#### Analysis and modification procedures after the interviews

The interviews conducted with the clinicians and older adults were audio recorded to ensure an accurate and comprehensive capture of all feedback provided (Beatty & Willis, 2007). In addition to quantitative data on perceived difficulty, a qualitative content analysis was carried out to evaluate participants’ comments and suggestions in depth.

Following each interview, one investigator carefully re-listened to the audio recordings. All verbal comments related to the comprehension, clarity, coherence, and applicability of each item were transcribed verbatim. These comments were systematically categorized in a table, linking each piece of feedback to the specific item, scoring option or instruction to which it referred.

The analysis followed a manual, item-by-item coding strategy in which: (1) each comment was classified according to the type of issue it addressed (*e.g.*, substitute a word for another, provide a certain type of specification, include examples, the question is long, *etc*.); (2) Comments were further assessed for relevance and frequency.

Although no qualitative software was used due to manageable volume of data, this structured procedure allowed for transparent tracking of participant input and supported decisions about item refinement.

In parallel, the quantitative indicators of perceived difficulty were analyzed using frequency distributions. Specifically, the frequency of responses indicating higher difficulty levels (*i.e.,* “very difficult,” “quite difficult,” and “somewhat difficult”) was examined for the following key aspects:

• Level of difficulty in calculating the total score (clinicians): High levels of difficulty in calculating the total score would suggest the need for simplification or clarification of the scoring procedure to ensure practical implementation.
• Level of difficulty in comprehending the task/question (older adults): Difficulty in understanding specific tasks or questions would necessitate modifications to ensure that the scale items are accessible and understandable for older adults, promoting accurate and meaningful responses.
• Level of difficulty in understanding response options (older adults): Similar attention was given to difficulties reported in understanding response options, with the goal of refining these options to make them more intuitive and aligned with the cognitive abilities of older adults.

Based on this comprehensive analysis, a list of proposed modifications was generated. This list included detailed descriptions of the comments received, and it highlighted which suggestions were deemed appropriate for incorporation into the revised version of the scale. The modifications were carefully deliberated to maintain the scale’s psychometric integrity while enhancing its practical utility.

Following these refinements, the third and final version of the scale, named EMFRA-P3, was developed. This version reflects the iterative improvements made to enhance both content validity and usability. To facilitate transparency, the changes made to the scale are highlighted in the Supplementary Material, providing a clear account of the modifications undertaken.

## Results

### Original development of the evaluation of multidimensional functioning and risks in aging scale

The items selected for the initial version of the scale included the following assessment domains: physical functioning, cognitive functioning, emotional status, and social features. A total of six items per domain were originally implemented, including both assessment tasks and self-reported items.

The physical functioning domain included two assessment tasks, corresponding to hand-grip strength and gait speed; as well as four self-reported questions, including gait assistance, balance, sedentarism, and fatigue.

The cognitive functioning domain included three assessment tasks, exploring language, arithmetic for money management, and attention-inhibition; and three self-reported questions concerning memory, concentration, and realization of cognitive activities.

The emotional status domain included self-reported questions assessing loneliness, sadness, fear, irritability, self-efficacy, and life satisfaction.

The social status domain included self-reported items of economic support, family interaction, social interaction, participation in ludic and social activities, communication ability, and receiving help.

Three categories for response and scoring were developed based on available literature cut-offs (for hand-grip strength, gait speed, and sedentarism). A three-category Likert scale for frequency, difficulty, and satisfaction. This three-category would be quantified with 0, 1 and 2 points respectively, indicating higher function in each domain.

EMFRA-P1 including item, response options and scoring methods can be seen Supplementary Material. Table 1 presents the selected items identified through the literature review, representing physical, cognitive, emotional, and social factors that have been associated, either longitudinally or cross-sectionally, with health-related adverse events. These potential risk factors are organized according to their respective content domains. For each item, the table summarizes the rationale for inclusion, highlighting key components of the item and the specific health-related deficits they are linked to.

**Table 1:** Justification for inclusion of items and response/punctuation options for EMFRA-P1.

**Item**	**Justification and synthesis of literature**
**Physical functioning**	
Item no. 1. Hand-grip strength	Two separate studies determined the optimal cut-off values for detecting mortality risk in older adults. In the ELSA cohort, the optimal cut-off values for males and females were found to be ≥36 kg and ≥23 kg, respectively. These values indicate higher mortality risk after a 14-year follow-up for individuals aged 60 or older (Spexoto et al., 2022). However, in the KORA cohort study, different optimal cut-off values were identified for males (≥29 kg) and females (≥18 kg), indicating mortality risk after 3 and 6 years of follow-up for older adults aged 65–93 (Huemer et al., 2023). Both studies followed a similar measurement protocol, which involved grip strength measurements taken from the dominant arm while seated, with the arm adducted along the trunk, elbow flexed at 90°, neutral prone-supination, and slight wrist extension. Three attempts were measured, taking the highest value. However, the study by Huemer et al. (2023) also included measurements while seated if standing was not possible. Although some research suggests no significant difference in grip strength measurements between standing and sitting positions, sitting may be more practical and comfortable for a broader range of older adults. Neither study mentioned resting the dynamometer on the thigh, which could potentially affect measurements. Rest time varied between the two studies, with only Spexoto et al. (2022) specifying a 1-minute rest period. Commonly used rest times range from 30–60 s (Sousa-Santos & Amaral, 2017). To ensure optimal recovery, a 1-minute rest period will be employed. Additionally, although neither study indicated a specific duration for each attempt, a range of 3 to 6 s will be used based on protocol recommendations (Sousa-Santos & Amaral, 2017). Verbal commands to encourage maximum force were not mentioned in either study and will not be used in the current test. Huemer et al. (2023) mentioned “The handle of the dynamometer was adjusted to fit the hand of the participants”, but did not specify an optimal position. Based on previous studies and experience, it is believed that the second handle position, or the most comfortable position is the one for achieving the greatest hand-grip force (Roberts et al., 2011).
Item no. 2. Gait speed	The study by Spexoto et al. (2022) investigated normal walking speed using a 2.4-meter linear path, following the procedures of the linear walk task of the Short Physical Performance Battery (Guralnik et al., 1994). The researchers recorded the best value, indicating the shortest time, from two consecutive attempts. Although the authors did not specify the rest time between attempts, they did specify that participants should perform the test without assistive walking devices. However, this requirement has not been included in the present scale, as we desired assessing a broader range of patients. Furthermore, the use of assistive walking devices will be addressed with the following items. Additionally, this item assesses the inability to walk, as previous studies have observed that patients unable to walk present a higher risk of mortality at 3-month follow-up after hospital discharge (Pasina et al., 2018) and present higher levels of sarcopenia, compared to those able to walk (Maeda et al., 2017).
Item no. 3. Gait assistance	The use of gait assistive devices is associated with health-related impairments. For example, among older adults aged 70 or above, the use of these devices predicts mortality risk at a 3-year follow-up (Gray et al., 2016). Similarly, a meta-analysis involving older adults aged 65 and above who underwent knee or hip arthroplasty found that using such devices is associated with a heightened risk of falls (Liu et al., 2020). Additionally, another meta-analysis focusing on older adults residing in nursing homes or those hospitalized revealed that the use of assistive walking devices is correlated with an increased risk of falls (Deandrea et al., 2013).
Item no. 4. Balance	Findings from a systematic review reveal that among older adults admitted to emergency departments, reporting a “sensation of loss of balance” exhibits a sensitivity of 61% and specificity of 64% in detecting fall risk at a 6-month follow-up (Carpenter et al., 2014). Furthermore, data from a meta-analysis indicate that loss of balance is also associated with poorer overall cognitive status (Demnitz et al., 2016). Another meta-analysis suggests that loss of balance longitudinally predicts fall risk in older adults (Jehu et al., 2021). Moreover, a systematic review and meta-analysis demonstrate that loss of balance longitudinally predicts the risk of disability in activities of daily living among older adults (Vermeiren et al., 2016; Wang et al., 2020).
Item no. 5. Sedentarism	From a meta-analysis in middle-aged and elderly adults, it was determined that 9 h or less per day is a protective factor on the risk of mortality (upper limit of the confidence interval in HR < 1), if it was higher than 9 h per day the subjects are at risk of mortality (lower limit of the confidence interval in HR > 1) and when ≥ 11 hours/day the HR for risk of mortality is equal or higher than 1.75), observed in a follow-up of between 4 and 8.9 years. This data was extracted from Fig. 2 of Ekelund et al. (2019).
Item no. 6. Fatigue	Based on data from a meta-analysis (Knoop et al., 2021) it has been determined that fatigue in older adults, whether during activities or daily living, increases the risk of all-cause mortality in a follow-up period from 3 to 15 years (OR: 2.29 [1.67–3.14]; HR/RR: 1.47 [1.34–1.61]), being higher among those with moderate (HR/RR: 1.22 [1.09–1.36]) and high levels of fatigue (HR/RR: 1.36 [1.19–1.55]). Moreover, older adults with fatigue also present a risk of mortality associated with cardiovascular issues during a follow-up of 12 to 20 years (HR/RR: 1.38 [1.06–1.81]). The same same meta-analysis also reveals a heightened risk of hospitalization among older adults experiencing fatigue, with a follow-up period ranging from 5 to 16 years ((HR: 1.74 [1.48–2.05]). Furthermore, this risk increases with the severity of fatigue, with those experiencing mild (HR/RR: 1.28 [1.13–1.45]), moderate (HR/RR: 1.43 [1.24–1.65]) and high (HR/RR: 1.69 [1.36–2.09]) levels of fatigue showing increased rates of hospitalization. Fatigue is also associated with higher risk of limitations and declines in physical function during a follow-up period of 1.5 to 8 years (OR: 1.41 [1.58–4.08]). Additionally, it increases the likelihood of experiencing high levels of disability in both in BADL and IADL during a follow-up of 5 to 10 years (HR: 3.70 [2.80–4.87]).
**Cognitive functioning**	
Item no. 7. Language	Although we haven’t come across a dedicated reference for evaluating rhyming ability or phoneme counting, language-related tests are commonly incorporated in global cognitive function assessment tools, such as the Montreal Cognitive Assessment scale (Nasreddine et al., 2005). We consider these tests to be cognitively challenging and suitable for our purposes.
Item no. 8. Arithmetic for money management	In older aged 70 or above, increasing age as well as the presence of cognitive impairment predicts a higher risk of difficulties with money management over the long term (Reynolds & Silverstein, 2003). Older adults (≥60 years) with cognitive impairment show differences in performance in money management compared to those without cognitive decline. For example, the “Peruvian Coin Test” was utilized presents good properties for detecting cognitive impairment (Oscanoa et al., 2016).
Item no. 9. Attention-inhibition	This item assesses both sustained attention ability (accuracy in tapping the correct number) and inhibition ability (correctly avoiding tapping). Older adults (≥50 years) present significant associations between sustained attention tests and physical frailty status. Poorer performance on these tests correlates with higher levels of frailty (O’Halloran et al., 2014). Among cognitively impaired older adults, lower attention and working memory is moderately associated with increased disability in ADL (Martyr & Clare, 2012). Inhibition ability is associated to overall physical function, with those exhibiting better physical function also demonstrating superior performance on inhibition tests (Braga et al., 2022). Furthermore, a low performance on inhibition tests, such as the Stroop test, is identified as a risk factor for chronic pain occurrence during a 5-year follow-up among middle-aged and older adults (Stroop test interference index, OR: 1.49 (1.03–2.15) (Rouch et al., 2021).
Item no. 10. Memory	Based on findings from a meta-analysis, it has been determined that subjective cognitive impairment among older adults significantly increases the risk of dementia incidence (OR: 2.48, 95% CI [1.97–3.14]) and mild cognitive impairment (OR: 1.83, 95% CI [1.56–2.16]) (Pike et al., 2022). Subjective perception of short-term memory loss is associated with higher mortality risk in older adults aged 70–79 years at an average 8-year follow-up period (HR for mild subjective memory loss: 1.51, 95% CI [1.20–1.91]; HR for moderate or severe subjective memory loss: 1.62 95% CI [1.08–2.43]) (Tangen, Langballe & Strand, 2020). Furthermore, when the presence of subjective perception of short-term memory loss is combined with the presence of disability in activities of daily living, the risk of mortality is enhanced in older adults aged 70 years or older at a mean 8-year follow-up (HR in older adults aged 70–79 years: 2.63 95% CI [1.98–3.49]; HR in older adults ≥80 years: 1.40 95% CI [1.09–1.81]) (Tangen, Langballe & Strand, 2020). Similarly, the combined presence of subjective memory and low physical frailty in older adults (≥65 years is a risk factor for mortality at 5-year follow-up (HR: 1.48 95% CI [1.02–2.16]) (Li et al., 2023).
Item no. 11. Concentration	Based on findings from a meta-analysis, the presence of subjective cognitive impairment among older adults significantly increases the risk of dementia incidence (OR: 2.48 95% CI [1.97–3.14]) and mild cognitive impairment (OR: 1.83 95% CI [1.56–2.16]) (Pike et al., 2022). Specifically, difficulty or lack of concentration predicts the occurrence of mild cognitive impairment in older adults (≥55 years) at a 2-year follow-up (OR: 1.71 95% CI [1.17–2.50]) (Lobo et al., 2008).
Item no. 12. Cognitive activities	Engaging in frequent cognitive activities is associated with less cognitive impairment (Kurita et al., 2020). Many of these activities included in the item come from those proposed by Verghese et al. (2003) who developed a scale in itinerary format to assess various cognitive activities. They observed that a higher frequency of cognitive activities in the scale protected subjects from dementia. Additionally, studies focusing on reading habits have observed that higher reading-related skills are associated with enhanced episodic memory (Sol et al., 2021). Moreover, spending more hours reading is associated with overall cognitive function (Chino et al., 2022). Reading books, rather than magazines, seems to be protective on verbal fluency and episodic memory over a 15-year period (Sörman, Ljungberg & Rönnlund, 2018). A meta-analysis found that performing cognitive activities is reduces the risk of all-cause dementia, and, more specifically, the risk of Alzheimer’s disease (Su et al., 2022). Additionally, a cohort study observed that older adults (≥60 years) who engage in cognitive activities have a lower risk of all-cause mortality at 4-year follow-up (Liu et al., 2021).
**Emotional status**	
Item no. 13. Loneliness	Research shows that both subjective loneliness (feeling lonely) and objective factors (isolation, companionship, *etc*.) increase the risk of mortality in older adults (>60 years) over a period of 3 to 28 years (OR: 1.10 CI 95% [1.06–1.14]) (Schutter et al., 2022). Similarly, in middle-aged people (50–65 years), experiencing loneliness is associated with a higher likelihood of work disability in the medium term, and is associated with the presence of depression (Morris, 2020).
Item no. 14. Sadness	In older adults (≥50 years), experiencing sadness is associated with a more frequent contact with healthcare professionals in the previous year (OR: 1.10 95% CI [1.04–1.12]), a history of depressive symptoms (OR: 2.18 95% CI [1.78–2.67]), and prior admissions to psychiatric services (OR: 2.87 95% CI [1.76–4.12]) (Komulainen et al., 2021). Additionally, the presence of sadness combined with anhedonia in older adults (≥62 years) is a risk factor for the occurrence of disability or mortality over a 13-year period (OR: 1.28 95% CI [1.13–1.46]) (Covinsky et al., 2014).
Item no. 15. Fear	Although we have not found a specific reference for the use of this item, it is derived from a similar item found in the Pain Catastrophizing Scale. We consider that it may be useful to integrate it into the the EMFRA.
Item no. 16. Irritability	Older adults (≥70 years) who exhibit irritability have a higher risk of mild cognitive impairment (HR: 1.84 95% CI [1.31–2.58]) (Geda et al., 2014). Similarly, another study involving cognitively healthy older adults (55–90 years) found that the presence of irritability features increases the risk for dementia at a 4-year follow-up (HR: 3.65 95% CI [1.80–7.40]) (Jang et al., 2020). In addition, it is associated with social friction (Miloyan & Pachana, 2016) and psychotic symptoms (Östling et al., 2019).
Item no. 17. Self-efficacy	Among older adults, self-efficacy is positively associated with healthy aging (Wu & Sheng, 2019). Additionally, self-efficacy has been identified as a mediating factor in enhancing life satisfaction among frail older adults (Qin et al., 2020).
Item no. 18. Life satisfaction	Older adults (>50 years), with moderate to high levels of life satisfaction experienced a reduced risk of all-cause mortality, chronic illnesses, physical limitations, depressive symptoms, and loneliness at a 4-year follow-up (Kim et al., 2021).
**Social status domain**	
Item no. 19. Economical support	Family financial support serves as a protective factor in older adults, reducing the risk of developing depressive symptoms over a 2 to 5 years follow-up period (Cai et al., 2023). Moreover, among older adults (>60 years), higher levels of financial support from their children is associated with better self-perceived health over a 2 to 8 year follow-up (Liu et al., 2015).
Item no. 20. Family interaction	A consistent family network is associated with increased life satisfaction in adults (Cheng et al., 2022). Moreover, older adults (60–85 years) who regularly meet or see their shildren have a lower risk of depressive symptoms over a 2–5-year follow-up period (OR medium frequency: 0.86 95% CI [0.78–0.99]; OR high frequency: 0.77 95% CI [0.69–0.86]) (Cai et al., 2023).
Item no. 21. Social interaction	A consistent family network is associated with increased life satisfaction in adults (Cheng et al., 2022).
Item no. 22. Participation in ludic and social activities	Older adults (>65 years) with greater participation in social activities present lower levels of anxiety and loneliness, and higher cognitive functions (Li et al., 2021).
Item no. 23. Communication ability	Although we have not found a specific reference for the use of this item, we consider it to be relevant for its possible implication in isolation, and sense of belonging to the community. The lack of communication skills through digital media may limit social interactions in the elderly, and may even indicate losses related to cognitive functions.
Item no. 24. Receiving help	Social support among older adults is positively associated with their perceived quality of life (Moghadam et al., 2020). Moreover, older adults who have strong social support networks are likely to present lower depressive symptoms (Gariépy, Honkaniemi & Quesnel-Vallée, 2016).

### Content validity by experts

A total of 15 Spanish experts participated in the content validation panel, with a mean age of 42 years (range: 27–57). Most resided in Madrid, with two based in the Canary Islands. The panel was multidisciplinary, including physiotherapists (most frequent), physicians, psychologists, occupational therapists, podiatrists, and exercise professionals. Their professional backgrounds spanned academia, clinical practice (both public and private), research, and health policy. Regarding education, 10 held a PhD, two were predoctoral researchers, two held master’s degrees, and one held a bachelor’s degree. More information concerning sociodemographic and professional background is provided in Table 2.

**Table 2:** Experts’ descriptive data for EMFRA content validation.

	**Mean ± SD**	**Min**	**Q1**	**Median**	**Q3**	**Max**
**Age (years)**	42.13 ± 10.23	27	33.5	42	51.5	57
**Sex (*n*, %)**						
Male ∣ Female	8, 53.3% | 7, 46.7%					
**Nationality (*n*, %)**						
Spanish	15, 100%					
**Native language (*n*, %)**						
Spanish from Spain	15, 100%					
**Country of residence (*n*, %)**						
Spain	15, 100%					
**Autonomous community of residence (*n*, %)**						
Madrid ∣ Canary Islands	13, 86.7% ∣ 2, 13.3%					
**Educational level (*n*, %)**						
PhD — Predoctoral student	10, 66.7% — 2, 13.3%					
Master — Bachelor	2, 13.3% ∣ 1, 6.7%					
**Profession (*n*, %)**						
Physiotherapist	6, 40.0%					
Physiotherapist, and Exercise professional	1, 6.7%					
Physiotherapist, and Physician	1, 6.7%					
Physiotherapist, and Occupational therapist	2, 13.3%					
Podiatrist	1, 6.7%					
Psychologist	1, 6.7%					
Occupational therapist	3, 20.0%					
**Employment status (*n*, %)**						
University teaching staff	3, 20.0%					
University teaching staff, and Research staff	5, 33.3%					
University teaching staff, Research staff, and Political staff	1, 6.7%					
University teaching staff, Research staff, and Public healthcare service	1, 6.7%					
University teaching staff, Research staff, and Private healthcare service	1, 6.7%					
University teaching staff, Research staff, and Public and Private healthcare service	1, 6.7%					
University teaching staff, and Private healthcare service	1, 6.7%					
Public healthcare service	1, 6.7%					
Private healthcare service	1, 6.7%					
**Clinical practice experience with older adults (years)**	10.13 ± 5.62	0	5.5	10	13	20
**Research experience with older adults (years)**	5.07 ± 4.50	0	0	5	8	14
**Clinical practice experience with frail populations (years)**	7.87 ± 5.54	0	4	8	11	20
**Research experience with frail populations (years)**	3.93 ± 3.51	0	0	4	7	9
**Clinical practice experience with measurement tools (years)**	9.07 ± 4.56	2	6.5	10	10	20
**Research experience with measurement tools (years)**	6.40 ± 3.87	0	3.5	7	9.5	13
**Clinical practice experience with frailty measurement tools (years)**	8.13 ± 5.54	0	5	10	11	20
**Research experience with frailty measurement tools (years)**	4.33 ± 3.77	0	0	4	7	10
**Current participation in other projects with frail older adults (*n*, %)**						
Yes — No	6, 40.0% — 9, 60.0%					
**Previous participation in content validation processes (n)**	1.87 ± 2.03	0	0	1	3.5	5

**Figure 1:**
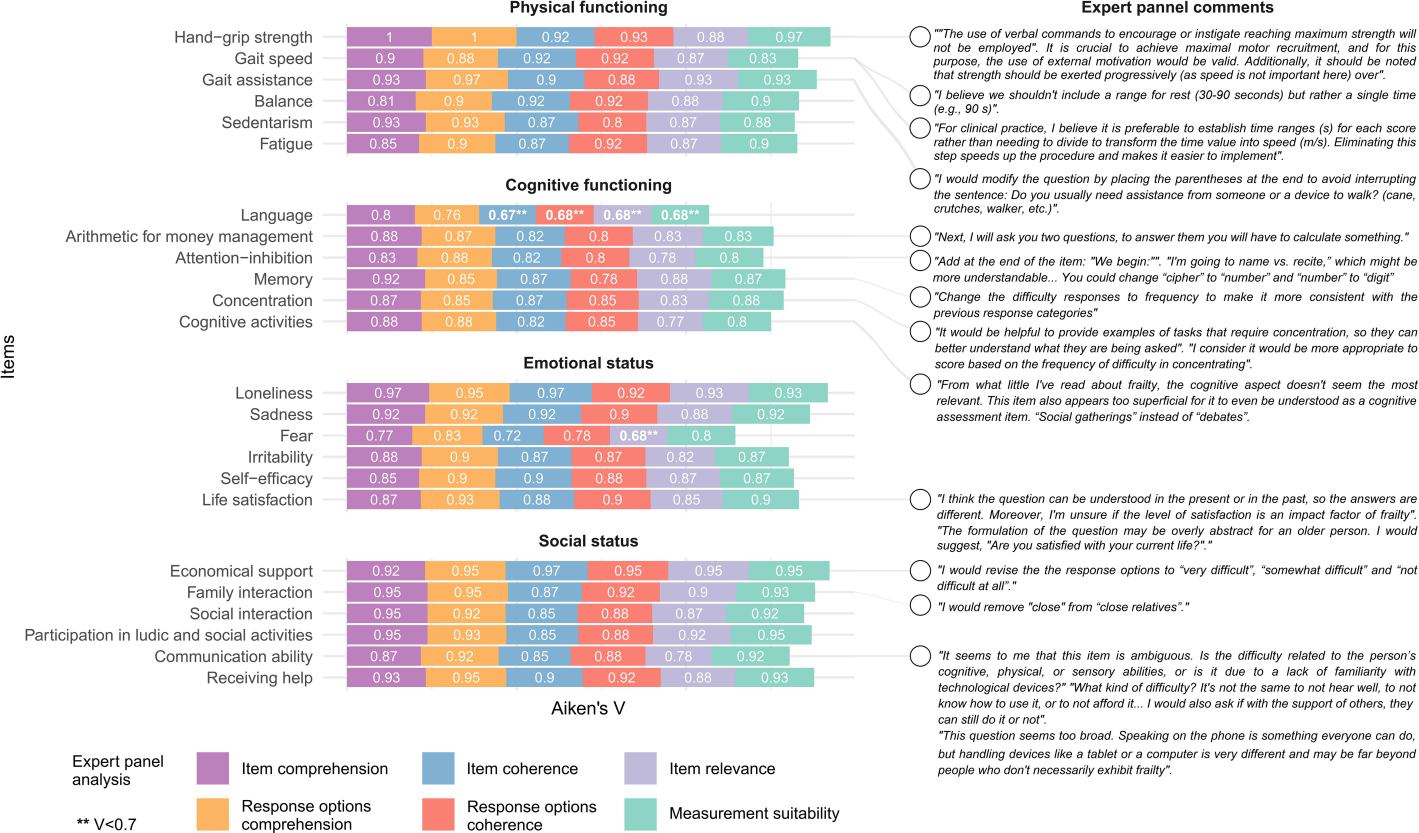
Aiken’s V and expert comments.

The level of agreement in Aiken’s V ranged from 0.67 to 1. A total of 22 items presented exceeded *V* > 0.7 in all the assessment domains. Only item no. 7 (Language), and item 15 (Fear) presented oner or more domains of agreement of *V* < 0.7. These two items were excluded due to low agreement, whereas the others were maintained and modified based on the frequency and suitability of retrieved comments. Aikens’ V for each assessment domain and relevant authors’ comments highlighting the ones considered for EMFRA modification can be seen in Fig. 1. EMFRA-P2 is presented with its respective changes in Supplementary Material.

### Content validity by clinicians and older adults

A total of 10 clinicians residing in Madrid (Spain) were interviewed. All had a native or proficiency level in Spanish. Their educational backgrounds included PhD, predoctoral student, master’s and bachelor’s degrees. Most were physiotherapists, along with two podiatrists. Their employment status included university teaching, research, private healthcare, and domiciliary attention. Clinical experience with older adults ranged from 0 to 10 years, while experience using assessment tools varied from 0.2 to 8 years. Additional demographic and professional details are presented in Table 3.

Twelve older adults were initially recruited, but only 10 were interviewed, as two were excluded due to low MoCA scores (<26 points). The cognitive scores of the final participants ranged from 27 to 30. Their educational backgrounds included high school, professional training, and university studies, with a total year of education ranging from 18 to 27. Their employment status varied, with most being retired, active workers, or domestic workers. Additional sociodemographic information is presented in Table 4.

**Figure 2:**
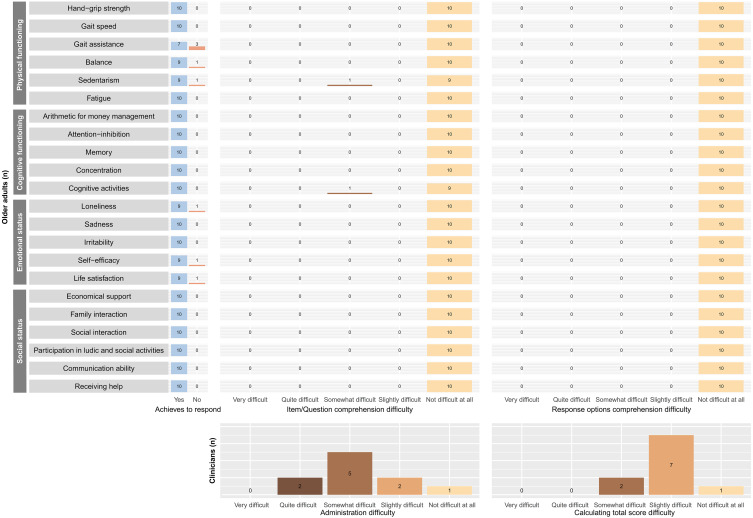
Cognitive interviews quantitative results.

The clinicians more frequently reported a difficulty level of “somewhat difficult” for administering the EMFRA-P2, and “slightly difficult” for calculating the total scale score. Furthermore, the quantitative analysis revealed that all older adults were able to completely comprehend all EMFRA-P2 items and its response options. There was only one item (“cognitive activities”) for which an older adult reported a “somewhat difficult” level of difficulty for its comprehension. All the older adults provided a response for all items, except for items no. 3 (gait assistance), no. 4 (balance), no. 5 (sedentarism), no. 13 (loneliness), no. 17 (self-efficacy), and no. 18 (life satisfaction). The motive for not being able to provide a response was derived from the absence of response categories that completely represented the status of the participant. In this case, the motive was the absence of a category of response such as “never” for items no. 3, 4, 13, 17, and 18. The motive for not achieving a response for item no. 5 was the inability to mentally remember and calculate the amount of sedentary time. All this information is provided in Fig. 2.

**Table 3:** Clinicians descriptive data for EMFRA-P2 content validation.

	**Mean ± SD**	**Min**	**Q1**	**Median**	**Q3**	**Max**
**Age (years)**	26.4 ± 3.95	22	23	25.5	28.75	23
**Sex (*n*, %)**						
Male — Female	7, 70% — 3, 30%					
**Nationality (*n*, %)**						
Spanish	9, 90%					
Italian	1, 10%					
**Native language (*n*, %)**						
Spanish from Spain	9, 90%					
Italian	1, 10%					
**Spanish from spain language level (*n*, %)**	
Native	9, 90%					
C2	1, 10%					
**Country of residence (*n*, %)**						
Spain	10, 100%					
**Autonomous community of residence (*n*, %)**						
Madrid	10, 100%					
**Educational level (*n*, %)**						
PhD — Predoctoral student	1, 10% — 3, 30%					
Master — Bachelor	3, 30% ∣ 3, 30%					
**Profession (*n*, %)**						
Physiotherapist	7, 70%					
Physiotherapist, and Informatitian	1, 10%					
Physiotherapist, and Podiatrist	1, 10%					
Podiatrist	1, 10%					
**Employment status (*n*, %)**						
University teaching staff	1, 10%					
University teaching staff, and Private healthcare service	2, 20%					
University teaching staff, Private healthcare service, and Research staff	1, 10%					
Private healthcare service	1, 10%					
Private healthcare service, and Domiciliary attention	2, 20%					
Public health system, and Domiciliary attention	1, 10%					
Unemployed	2, 20%					
**Clinical practice experience (years)**	4.36 ± 4.09	0.3	0.78	3	7.5	11
**Clinical practice experience with older adults (years)**	2.4 ± 3.3	0	0.08	0.85	3	10
**Clinical practice experience in other frail populations (years)**	2.28 ± 2.68	0	0.03	1.85	3	8
**Clinical experience using tests and evaluation procedures (years)**	3.05 ± 2.88	0.2	0.78	2.5	3.75	8

**Table 4:** Older adults’ descriptive data for EMFRA-P2 content validation.

	**Mean ± SD**	**Min**	**Q1**	**Median**	**Q3**	**Max**
**Age (years)**	70.0 ± 5.06	65	66	69	72	79
**Sex (*n*, %)**						
Male — Female	4, 40% — 6, 60%					
**Height (m)**	1.65 ± 0.07	1.57	1.59	1.64	1.7	1.76
**Weight (kg)**	71.8 ± 14.14	58	60.75	69.5	78.75	103
**BMI (kg/m**^**2**^)	26.3 ± 3.74	21.8	23.7	25.5	28.1	33.3
**MoCA**	28.3 ± 0.95	27	28	28	29	30
**Completed educational level**						
High school	1, 10%					
Professional training	2, 20%					
University	7, 70%					
**Studied years**	20.9 ± 3.96	15	18	20.5	23.8	27
**Marital status**						
Divorced	2, 20%					
Married	8, 80%					
**Laboral status**						
Unpaid domestic worker	1, 10%					
Active	2, 20%					
Retired	7, 70%					

The most relevant and frequent comments from the clinicians and older adults are summarized in Table 5.

**Figure 3:**
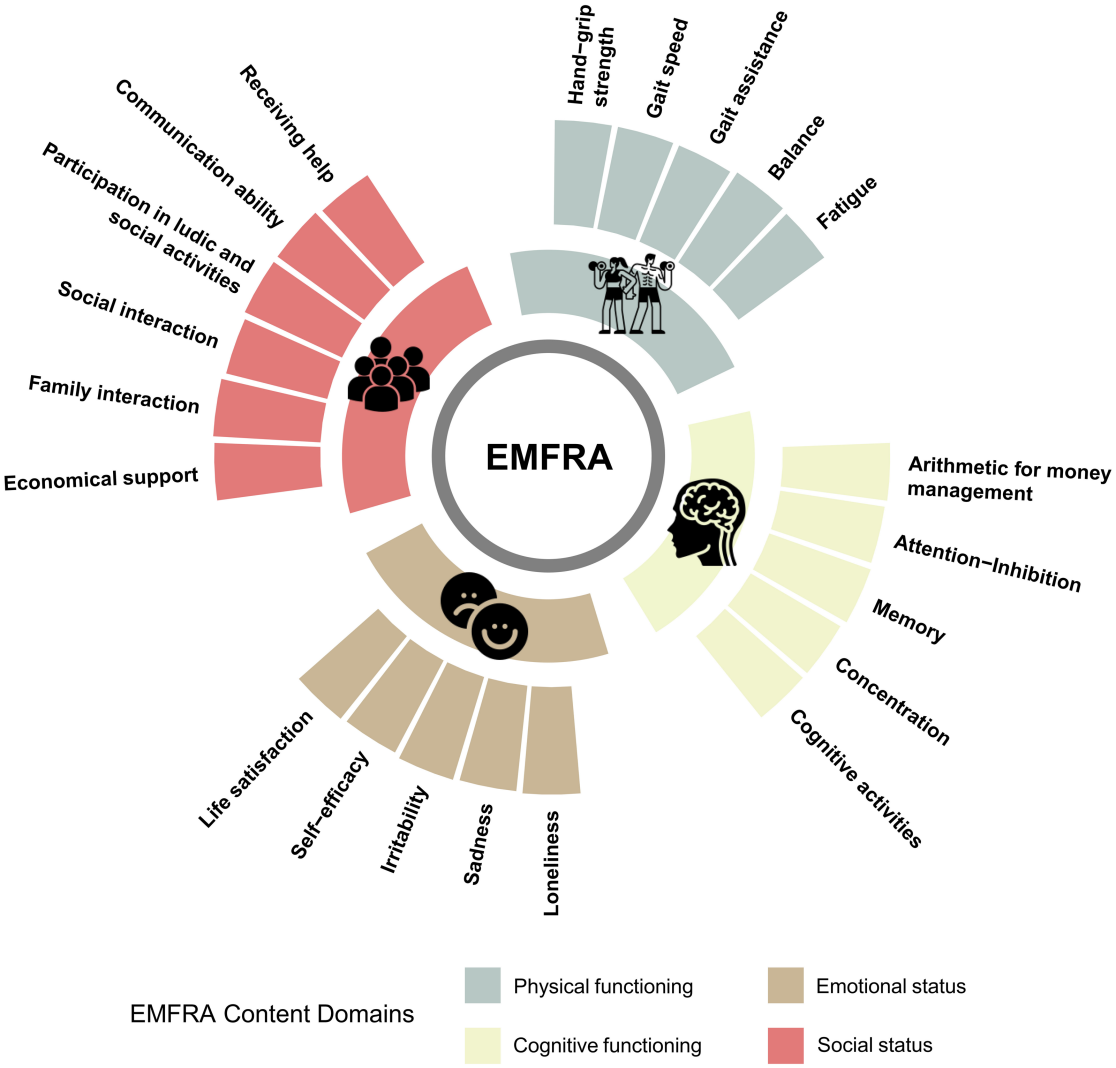
EMFRA content domains after content validation process.

Based on the clinicians’ comments and the older adults’ inability to calculate sedentary time, the authors decided to exclude item no. 5 (sedentarism) due to the validity of the measurement procedure (self-reported). Modifications were conducted based on the frequency and relevance of commentaries for developing the EMFRA-P3. Highlighted modifications are indicated Supplementary Material. The final clean original version (in Spanish) of EMFRA-P3 and its translated version to English can be seen in Supplementary Material. A summary of EMFRA-P3 content domains can be found in Fig. 3.

## Discussion

The present study aimed to develop and validate a new tool, the EMFRA scale, specifically designed to assess multidimensional functioning and risks in older adults in a Spanish-speaking context. The EMFRA addresses the level of functioning by incorporating domains that address physical health, including cognitive, emotional, and social dimensions. This perspective on functioning aligns with contemporary understandings of the health within the biopsychosocial framework and the ICF, which emphasize the interaction between individuals and their environment as key determinants of health status (World Health Organization, 2001).

The inclusion of multiple domains in the assessment of functioning, as achieved with the EMFRA, reflects growing evidence that health is not limited to physical functioning alone but also involves cognitive and psychosocial factors. This is supported by recent findings indicating that cognitive impairment and social isolation significantly contribute to negative health outcomes in older adults, including reduced quality of life and increased mortality risk (Dent et al., 2019). By incorporating these additional dimensions, EMFRA provides a more holistic and domain-specific evaluation of functioning, potentially improving the identification of individuals at risk of adverse outcomes and allowing for more targeted interventions.

Our approach to content validation, involving both healthcare experts and target populations, has helped ensure that the EMFRA is both comprehensive and user-friendly. This methodological strategy addresses key limitations observed in other tools, particularly those focused on frailty, which often lack clarity, theoretical grounding, and clinical interpretability (Fierro-Marrero et al., 2025). In line with COSMIN recommendations, the inclusion of end-users (clinicians and older adults) in the development process improved the instrument’s clarity, usability, and contextual relevance (De Vet et al., 2011). The feedback obtained through cognitive interviews led to significant refinements in item clarity and response options.

Although EMFRA shares with frailty instruments a pragmatic utility as a screening tool, its underlying conceptual model differs. Rather than relying on the ambiguous and variable defined construct of frailty, EMFRA adopts a domain-based approach to assess specific areas of functioning. This distinction allows clinicians to detect which domains are preserved and compromised, thus improving clinical decisions. In addition, the conceptual model of EMFRA could help clinicians screening functioning limitations before providing further profound evaluations withing the CGA (Brown et al., 1988).

### Study limitations

Despite these strengths, there are several limitations to consider. The content validation process involved a panel of 15 experts, which aligns with recommended standards for quantitative evaluation of content validity (Lynn, 1986). However, the cognitive interviews were conducted with 10 clinicians and 10 older adults, and while this sample size is methodologically accepted for identifying major comprehension and clarity issues (Beatty & Willis, 2007), it may not be sufficient to uncover all potential weaknesses in the scale (Blair et al., 2006). However, our use of a diverse panel of experts and the inclusion of both clinicians and older adults in cognitive interviews aimed to address potential biases and enhance the scale’s relevance across different user perspectives.

**Table 5:** Relevant comments extracted from the cognitive interview to clinicians and older adults.

**Commentaries**	**Clinicians**		**Older adults**	
	**Item**	**Responses**	**Item**	**Responses**
		**Physical functioning**		
Item no. 1. Hand-grip strength	*Differentiate between tests and hetero-completed items, using italics and quotation marks.”Change “attempts” to “trials”.*	*NCS*	*NCS*	*NCS*
Item no. 2. Gait speed	*Include examples of walking assistive devices.*	*NCS*	*NCS*	*NCS*
Item no. 3. Gait assistance	*Put the examples after the question.*	*Include the categories “always” and “never”. “Almost always” is similar to “sometimes”.*	*Start the examples with “such as...”.*	*Include the categories “always” and “never”.*
Item no. 4. Balance	*Include examples.*	*Include the categories “always” and “never”.*	*NCS*	*Include the categories “always” and “never”.*
Item no. 5. Sedentarism	*Specify that it evaluates during the daytime period. Maybe not valid through self-report.*	*NCS*	*Specify that it evaluates the daytime period and “in a usual day”.*	*NCS*
Item no. 6. Fatigue	*Specify “on a typical day”. Include the feminine term “tired”.*	*Include the categories “always” and “never”. “Almost always” is similar to “sometimes”.*	*Specify “in a usual day”*	*Include the categories “always” and “never”.*
		**Cognitive functioning**		
Item no. 7. Arithmetic for money management	*Change “would receive” to “will receive”. Replace “article” with “product”.*	*NCS*	*NCS*	*NCS*
Item no. 8. Attention-inhibition	*Use only one registry (3*rd *or 1*st *person). Use a stopwatch instead of a metronome. Do not use words in diminutive form.*	*Simplify and condense the scoring categories.*	*NCS*	*NCS*
Item no. 9. Memory	*Replace “recent” with a specific time frame.*	*Include the categories “always” and “never”. “Almost always” is similar to “sometimes”.*	*“Recent events” and “daily tasks” are very different to remember. Keep only one.*	*Include the categories “always” and “never”.*
Item no. 10. Concentration	*Include examples and a specific time.*	*Include the categories “always” and “never”. “Almost always” is similar to “sometimes”.*	*NCS*	*Include the categories “always” and “never”.*
Item no. 11. Cognitive activities	*The question is too long. Specify that it evaluates the most frequent occurrence of at least 1 activity. Move the examples after finishing the question. Reduce the number of examples. Replace “reading books, magazines, or newspapers” with “reading”.*	*Include the categories “always” and “never”. Modify the response options to indicate the number of days per week.*	*The question is too long. Specify that it assesses the highest frequency of at least 1 activity. Change “organized group debates” to “social gatherings”*	*Include the categories “always” and “never”.*
		**Emotional status**		
Item no. 12. Loneliness	*Include “in a usual week”.*	*Include the categories “always” and “never”. “Almost always” is similar to “sometimes”.*	*NCS*	*Include the categories “always” and “never”.*
Item no. 13. Sadness	*Include “in a usual week”.*	*Include the categories “always” and “never”. “Almost always” is similar to “sometimes”.*	*NCS*	*Include the categories “always” and “never”. Modify “Almost always” for “sometimes”.*
Item no. 14. Irritability	*Include “in a usual week”.*	*Include the categories “always” and “never”. “Almost always” is similar to “sometimes”.*	*NCS*	*NCS*
Item no. 15. Self-efficacy	*Change “stressful” to “difficult”.*	*Include the categories “always” and “never”.*	*NCS*	*NCS*
Item no. 16. Life satisfaction	*Change “with life” to “with your life”. Reframe the question as follows: “If you analyse your day-to-day life over the past few weeks, how satisfied do you feel?”*	*Include “not satisfied at all”*	*NCS*	*Change “very dissatisfied” to “not satisfied at all”.*
		**Social status**		
Item no. 17. Economical support	*Change “economic support” to “economic situation”. Modify the question as follows: “With your current economic situation, are you able to...”. Place the examples after the question.*	*The Likert scale feels odd. Keep the Likert scale but modify the question to “Do you find it difficult to satisfy...?”. If not modifying the item, change the Likert scale to categories of “ability” or alternatively respond based on “frequency”.*	*The question is too long. Start the examples with “such as...”.*	*The Likert scale feels, please modify it.*
Item no. 18. Family interaction	*NCS*	*Include the categories “always” and “never”. “Almost always” is similar to “sometimes”.*	*NCS*	*Include the categories “always” and “never”.*
Item no. 19. Social interaction	*Include examples*	*Include the categories “always” and “never”.*	*NCS*	*NCS*
Item no. 20. Participation in ludic and social activities	*Include examples*	*Include the categories “always” and “never”.*	*Include examples*	*“Almost never” is an odd term, consider modifying it.*
Item no. 21. Communication ability	*Change the focus of the question to: “Do you see yourself capable of communicating…?”*	*Include the categories “always” and “never”.*	*NCS*	*Include the category “never”.*
Item no. 22. Receiving help	*Include examples. Change the focus of the question to: “If you had any problem, do you have anyone who could help you?”*	*Include the categories “always” and “never”.*	*The question is confusing and not well understood.*	*Include the category “never”.*

**Notes.**

NCS, no comments suggested.

### Future research directions

Future research should aim to further evaluate the psychometric properties of the EMFRA, including its reliability and concurrent and predictive validity, in larger and more diverse populations. At its current stage of development, the scale cannot yet be recommended for clinical use until additional validation steps are completed.

Specifically, it is necessary to determine the structural validity of the scale to empirically confirm the presence of its four theoretical domains. This includes verifying whether all items adequately fit within their respective domains and identifying any that may require modification or removal due to poor alignment. The exploratory factor analysis should be conducted with a minimum sample size of 200–300 subjects (Lloret-Segura et al., 2014). To assess the suitability of the data for factor analysis, the Kaiser-Meyer-Olkin and Bartlett’s test of sphericity should be applied (Izquierdo, Olea & Abad, 2014). The number of factors to extract can be determined using a scree plot (Ferguson & Cox, 1993). An oblique rotation method, such as direct oblimin, is recommended to allow for potential correlations between factors. This exploratory analysis would provide an initial assessment of whether the four theoretical domains are supported by the data.

Additionally, concurrent validity should be assessed by comparing each EMFRA domain with existing gold-standard instruments that measure the corresponding construct. This will help determine whether the domains detected by EMFRA share meaningful variance with validated measures of physical function, cognitive function, emotional state and social domain.

It is also essential to establish test-retest reliability, ideally at the item and domain level, to confirm that the information captured by each item is both stable over time and sufficiently precise. These analyses will contribute to refining the instrument and confirming whether items should be maintained or removed.

Finally, to support clinical decision-making, it will be necessary to obtain prospective data on the predictive validity of EMFRA, to anticipate the occurrence of adverse health events in older adults, as has been done with other similar instruments (Vermeiren et al., 2016).

## Conclusions

The EMFRA scale represents a significant step forward in the assessment of functioning, particularly in Spanish-speaking populations in which there has been a lack of multidimensional tools. By integrating physical, cognitive, emotional, and social dimensions of functioning, the EMFRA provides a rapid and domain-based assessment that can enhance early identification of potential health risks in older adults and guide clinical decisions. The current state of EMFRA cannot be employed in clinical practice, as further psychometric properties must be determined.

##  Supplemental Information

10.7717/peerj.20108/supp-1 Supplemental Information 1 EMFRA preliminary version number 1 - before expert panel validation

10.7717/peerj.20108/supp-2 Supplemental Information 2 EMFRA preliminary version number 2 - after expert panel validation with highlighted changes

10.7717/peerj.20108/supp-3 Supplemental Information 3 EMFRA preliminary version number 3 - clean version translated in English

10.7717/peerj.20108/supp-4 Supplemental Information 4 EMFRA preliminary version number 3 - clean version Spanish original version

10.7717/peerj.20108/supp-5 Supplemental Information 5 EMFRA preliminary version number 3 - after clinicians and older adults validation with highlighted changes

10.7717/peerj.20108/supp-6 Supplemental Information 6 Clinicians validation data

10.7717/peerj.20108/supp-7 Supplemental Information 7 Experts validation data

10.7717/peerj.20108/supp-8 Supplemental Information 8 Older adults validation data

## Additional Information and Declarations

### Data Availability

The following information was supplied regarding data availability: The data is available in the Supplemental Files.

## References

[ref-1] Aburto JM, Villavicencio F, Basellini U, Kjærgaard S, Vaupel JW (2020). Dynamics of life expectancy and life span equality. Proceedings of the National Academy of Sciences of the United States of America.

[ref-2] Apóstolo J, Cooke R, Bobrowicz-Campos E, Santana S, Marcucci M, Cano A, Vollenbroek-Hutten M, Germini F, Holland C (2017). Predicting risk and outcomes for frail older adults: an umbrella review of frailty screening tools. JBI Database of Systematic Reviews and Implementation Reports.

[ref-3] Beatty PC, Willis GB (2007). Research synthesis: the practice of cognitive interviewing. Public Opinion Quarterly.

[ref-4] Blair J, Conrad F, Ackermann AC, Claxton G (2006). The effect of sample size on cognitive interview findings.

[ref-5] Braga PLG, Henrique JS, Almeida SS, Arida RM, Gomes da Silva S (2022). Factors affecting executive function performance of Brazilian elderly in the Stroop test. Brazilian Journal of Medical and Biological Research = Revista Brasileira De Pesquisas Medicas E Biologicas.

[ref-6] Brown AS, Brummel-Smith K, Burgess L, D’Agostino RB, Goldschmidt JW, Halter JB, Hazzard WR, Jahnigen DW, Phelps C, Raskind M, Schrier Jr RW, Sox HC, Williams SV, Wykle M (1988). National Institutes of Health Consensus Development Conference Statement: geriatric assessment methods for clinical decision-making. Journal of the American Geriatrics Society.

[ref-7] Cai Y, Qiu P, He Y, Wang C, Wu Y, Yang Y (2023). Age-varying relationships between family support and depressive symptoms in Chinese community-dwelling older adults. Journal of Affective Disorders.

[ref-8] Carpenter CR, Avidan MS, Wildes T, Stark S, Fowler SA, Lo AX (2014). Predicting geriatric falls following an episode of emergency department care: a systematic review. Academic Emergency Medicine : Official Journal of the Society for Academic Emergency Medicine.

[ref-9] Cheng W, Song W, Ye C, Wang Z (2022). Family networks, social networks, and life satisfaction of older adults in China. Healthcare (Basel, Switzerland).

[ref-10] Chino B, Zegarra-Valdivia J, de Frutos-Lucas J, Paredes-Manrique C, Custodio N (2022). Impact of Sociodemographic Features and Lifestyle on Cognitive Performance of Peruvian Adults. Journal of Alzheimer’s Disease.

[ref-11] Covinsky KE, Cenzer IS, Yaffe K, O’Brien S, Blazer) DG (2014). Dysphoria and anhedonia as risk factors for disability or death in older persons: Implications for the assessment of geriatric depression. The American Journal of Geriatric Psychiatry: Official Journal of the American Association for Geriatric Psychiatry.

[ref-12] Davis LL (1992). Instrument review: getting the most from a panel of experts. Applied Nursing Research.

[ref-13] Deandrea S, Bravi F, Turati F, Lucenteforte E, La Vecchia C, Negri E (2013). Risk factors for falls in older people in nursing homes and hospitals. A systematic review and meta-analysis. Archives of Gerontology and Geriatrics.

[ref-14] Demnitz N, Esser P, Dawes H, Valkanova V, Johansen-Berg H, Ebmeier KP, Sexton C (2016). A systematic review and meta-analysis of cross-sectional studies examining the relationship between mobility and cognition in healthy older adults. Gait & Posture.

[ref-15] Dent E, Morley JE, Cruz-Jentoft AJ, Woodhouse L, Rodríguez-Mañas L, Fried LP, Woo J, Aprahamian I, Sanford A, Lundy J, Landi F, Beilby J, Martin FC, Bauer JM, Ferrucci L, Merchant RA, Dong B, Arai H, Hoogendijk EO, Won CW, Abbatecola A, Cederholm T, Strandberg T, Gutiérrez Robledo LM, Flicker L, Bhasin S, Aubertin-Leheudre M, Bischoff-Ferrari HA, Guralnik JM, Muscedere J, Pahor M, Ruiz J, Negm AM, Reginster JY, Waters DL, Vellas B (2019). Physical frailty: ICFSR international clinical practice guidelines for identification and management. The Journal of Nutrition, Health & Aging.

[ref-16] DeVellis RF (2017). Scale development: theory and applications.

[ref-17] De Vet HCW, Terwee CB, Mokkink LB, Knol DL (2011). Measurement in medicine: a practical guide.

[ref-18] Ekelund U, Tarp J, Steene-Johannessen J, Hansen BH, Jefferis B, Fagerland MW, Whincup P, Diaz KM, Hooker SP, Chernofsky A, Larson MG, Spartano N, Vasan RS, Dohrn I-M, Hagströmer M, Edwardson C, Yates T, Shiroma E, Anderssen SA, Lee I-M (2019). Dose-response associations between accelerometry measured physical activity and sedentary time and all cause mortality: Systematic review and harmonised meta-analysis. The BMJ.

[ref-19] Engel GL (1977). The need for a new medical model: a challenge for biomedicine. Science.

[ref-20] Estefania CC, Zalazar-Jaime MF (2018). Entrevistas cognitivas: revisión, directrices de uso y aplicación en investigaciones psicológicas. Avaliacão Psicológica̧.

[ref-21] Faller JW, Pereira D do N, de Souza S, Nampo FK, Orlandi F de S, Matumoto S (2019). Instruments for the detection of frailty syndrome in older adults: a systematic review. PLOS ONE.

[ref-22] Ferguson E, Cox T (1993). Exploratory factor analysis: a users? Guide. International Journal of Selection and Assessment.

[ref-23] Fierro-Marrero J, Reina-Varona Á, Paris-Alemany A, La Touche R (2025). Frailty in geriatrics: a critical review with content analysis of instruments, overlapping constructs, and challenges in diagnosis and prognostic precision. Journal of Clinical Medicine.

[ref-24] Fried LP, Tangen CM, Walston J, Newman AB, Hirsch C, Gottdiener J, Seeman T, Tracy R, Kop WJ, Burke G, McBurnie MA (2001). Frailty in older adults: evidence for a phenotype. The Journals of Gerontology Series A: Biological Sciences and Medical Sciences.

[ref-25] Gariépy G, Honkaniemi H, Quesnel-Vallée A (2016). Social support and protection from depression: Systematic review of current findings in Western countries. The British Journal of Psychiatry: The Journal of Mental Science.

[ref-26] GBD 2019 Dementia Forecasting Collaborators (2022). Estimation of the global prevalence of dementia in 2019 and forecasted prevalence in 2050: an analysis for the Global Burden of Disease Study 2019. The Lancet. Public Health.

[ref-27] Geda YE, Roberts RO, Mielke MM, Knopman DS, Christianson TJH, Pankratz VS, Boeve BF, Sochor O, Tangalos EG, Petersen RC, Rocca WA (2014). Baseline neuropsychiatric symptoms and the risk of incident mild cognitive impairment: A population-based study. The American Journal of Psychiatry.

[ref-28] Ghasemi H, Kharaghani MA, Golestani A, Najafi M, Khosravi S, Malekpour M-R, Tabatabaei-Malazy O, Rezaei N, Ostovar A, Ghamari S-H (2025). The national and subnational burden of falls and its attributable risk factors among older adults in Iran from 1990 to 2021: findings from the global burden of disease study. BMC Geriatrics.

[ref-29] Gray WK, Dewhurst F, Dewhurst MJ, Orega G, Kissima J, Chaote P, Walker RW (2016). Rates and predictors of three-year mortality in older people in rural Tanzania. Archives of Gerontology and Geriatrics.

[ref-30] Gupta U (2013). Informed consent in clinical research: revisiting few concepts and areas. Perspectives in Clinical Research.

[ref-31] Guralnik JM, Simonsick EM, Ferrucci L, Glynn RJ, Berkman LF, Blazer DG, Scherr PA, Wallace RB (1994). A short physical performance battery assessing lower extremity function: association with self-reported disability and prediction of mortality and nursing home admission. Journal of Gerontology.

[ref-32] Haynes SN, Richard DCS, Kubany ES (1995). Content validity in psychological assessment: a functional approach to concepts and methods. Psychological Assessment.

[ref-33] Ho JY, Hendi AS (2018). Recent trends in life expectancy across high income countries: retrospective observational study. The BMJ.

[ref-34] Huemer M-T, Kluttig A, Fischer B, Ahrens W, Castell S, Ebert N, Gastell S, Jöckel K-H, Kaaks R, Karch A, Keil T, Kemmling Y, Krist L, Leitzmann M, Lieb W, Meinke-Franze C, Michels KB, Mikolajczyk R, Moreno Velásquez I, Pischon T, Schipf S, Schmidt B, Schöttker B, Schulze MB, Stocker H, Teismann H, Wirkner K, Drey M, Peters A, Thorand B (2023). Grip strength values and cut-off points based on over 200,000 adults of the German National Cohort—A comparison to the EWGSOP2 cut-off points. Age and Ageing.

[ref-35] Izquierdo I, Olea J, Abad FJ (2014). Exploratory factor analysis in validation studies: uses and recommendations. Psicothema.

[ref-36] Jang JY, Ho JK, Blanken AE, Dutt S, Nation DA (2020). Affective neuropsychiatric symptoms as early signs of dementia risk in older adults. Journal of Alzheimer’s Disease: JAD.

[ref-37] Jehu DA, Davis JC, Falck RS, Bennett KJ, Tai D, Souza MF, Cavalcante BR, Zhao M, Liu-Ambrose T (2021). Risk factors for recurrent falls in older adults: A systematic review with meta-analysis. Maturitas.

[ref-38] Junius-Walker U, Onder G, Soleymani D, Wiese B, Albaina O, Bernabei R, Marzetti E, ADVANTAGE JA WP4 Group (2018). The essence of frailty: a systematic review and qualitative synthesis on frailty concepts and definitions. European Journal of Internal Medicine.

[ref-39] Kim ES, Delaney SW, Tay L, Chen Y, Diener ED, Vanderweele TJ (2021). Life satisfaction and subsequent physical, behavioral, and psychosocial health in older adults. The Milbank Quarterly.

[ref-40] Knoop V, Cloots B, Costenoble A, Debain A, Vella Azzopardi R, Vermeiren S, Jansen B, Scafoglieri A, Bautmans I, Bautmans I, Bautmans I, Verté D, Beyer I, Petrovic M, De Donder L, Kardol T, Rossi G, Clarys P, Scafoglieri A, Cattrysse E, de Hert P, Jansen B (2021). Fatigue and the prediction of negative health outcomes: A systematic review with meta-analysis. Ageing Research Reviews.

[ref-41] Komulainen K, Gluschkoff K, García Velázquez R, Airaksinen J, Szmulewicz A, Jokela M (2021). Association of depressive symptoms with health care utilization in older adults: Longitudinal evidence from the Survey of Health, Aging, and Retirement in Europe. International Journal of Geriatric Psychiatry.

[ref-42] Kurita S, Tsutsumimoto K, Doi T, Nakakubo S, Kim M, Ishii H, Shimada H (2020). Association of physical and/or cognitive activity with cognitive impairment in older adults. Geriatrics & Gerontology International.

[ref-43] Li C-L, Stanaway FF, Chang H-Y, Chen M-C, Tsai Y-H (2023). Joint predictability of physical frailty/pre-frailty and subjective memory complaints on mortality risk among cognitively unimpaired older adults. European Journal of Ageing.

[ref-44] Li W, Sun H, Xu W, Ma W, Yuan X, Wu H, Kou C (2021). Leisure activity and cognitive function among Chinese old adults: The multiple mediation effect of anxiety and loneliness. Journal of Affective Disorders.

[ref-45] Liao X, Zuo L, Dong Y, Pan Y, Yan H, Meng X, Li H, Zhao X, Wang Y, Shi J, Wang Y (2022). Persisting cognitive impairment predicts functional dependence at 1 year after stroke and transient ischemic attack: a longitudinal, cohort study. BMC Geriatrics.

[ref-46] Liu X, Ruan Y, Huang L, Guo Y, Sun S, Chen H, Gao J, Shi Y, Xiao Q (2021). Cognitive leisure activity and all-cause mortality in older adults: A 4-year community-based cohort. BMC Geriatrics.

[ref-47] Liu H, Xiao Q, Cai Y, Li S (2015). The quality of life and mortality risk of elderly people in rural China: The role of family support. Asia-Pacific Journal of Public Health.

[ref-48] Liu Y, Yang Y, Liu H, Wu W, Wu X, Wang T (2020). A systematic review and meta-analysis of fall incidence and risk factors in elderly patients after total joint arthroplasty. Medicine.

[ref-49] Lloret-Segura S, Ferreres-Traver A, Hernández-Baeza A, Tomás-Marco I (2014). El análisis factorial exploratorio de los ítems: una guía práctica, revisada y actualizada. Anales de Psicología.

[ref-50] Lobo A, López-Antón R, de-la-Cámara C, Quintanilla MA, Campayo A, Saz P, ZARADEMP Workgroup (2008). Non-cognitive psychopathological symptoms associated with incident mild cognitive impairment and dementia, Alzheimer’s type. Neurotoxicity Research.

[ref-51] Lynn MR (1986). Determination and quantification of content validity. Nursing Research.

[ref-52] Maeda K, Shamoto H, Wakabayashi H, Akagi J (2017). Sarcopenia is highly prevalent in older medical patients with mobility limitation. Nutrition in Clinical Practice: Official Publication of the American Society for Parenteral and Enteral Nutrition.

[ref-53] Martyr A, Clare L (2012). Executive function and activities of daily living in Alzheimer’s disease: A correlational meta-analysis. Dementia and Geriatric Cognitive Disorders.

[ref-54] Meland E, Hjörleifsson S (2024). To reveal disease or to promote function –that is the question. Scandinavian Journal of Primary Health Care.

[ref-55] Miloyan B, Pachana NA (2016). Clinical Significance of Individual GAD Symptoms in Later Life. Journal of Geriatric Psychiatry and Neurology.

[ref-56] Mitnitski AB, Mogilner AJ, Rockwood K (2001). Accumulation of deficits as a proxy measure of aging. The Scientific World Journal.

[ref-57] Moghadam K, Mansour-Ghanaei R, Esmaeilpour-Bandboni M, Atrkar-Roshan Z (2020). Investigating the relationship between social support and quality of life in the elderly. Journal of Education and Health Promotion.

[ref-58] Morris ZA (2020). Loneliness as a predictor of work disability onset among nondisabled, working older adults in 14 countries. Journal of Aging and Health.

[ref-59] Nasreddine ZS, Phillips NA, Bédirian V, Charbonneau S, Whitehead V, Collin I, Cummings JL, Chertkow H (2005). The montreal cognitive assessment, MoCA: a brief screening tool for mild cognitive impairment. Journal of the American Geriatrics Society.

[ref-60] O’Halloran AM, Finucane C, Savva GM, Robertson IH, Kenny RA (2014). Sustained attention and frailty in the older adult population. The Journals of Gerontology. Series B, Psychological Sciences and Social Sciences.

[ref-61] Oscanoa TJ, Cieza E, Parodi JF, Paredes N (2016). [Evaluation of peruvian money test in screening of cognitive impairment among older adults]. Revista Peruana De Medicina Experimental Y Salud Publica.

[ref-62] Östling S, Bäckman K, Sigström R, Skoog I (2019). Is the prevalence of psychosis in the very old decreasing? A comparison of 85-year-olds born 22 years apart. International Journal of Geriatric Psychiatry.

[ref-63] Pasina L, Cortesi L, Tiraboschi M, Nobili A, Lanzo G, Tettamanti M, Franchi C, Mannucci PM, Ghidoni S, Assolari A, Brucato A, REPOSI Investigators (2018). Risk factors for three-month mortality after discharge in a cohort of non-oncologic hospitalized elderly patients: Results from the REPOSI study. Archives of Gerontology and Geriatrics.

[ref-64] Pike KE, Cavuoto MG, Li L, Wright BJ, Kinsella GJ (2022). Subjective Cognitive Decline: Level of Risk for Future Dementia and Mild Cognitive Impairment, a Meta-Analysis of Longitudinal Studies. Neuropsychology Review.

[ref-65] Qin W, Xu L, Sun L, Li J, Ding G, Wang Q, Zhang J, Shao H (2020). Association between frailty and life satisfaction among older people in Shandong, China: The differences in age and general self-efficacy. Psychogeriatrics: The Official Journal of the Japanese Psychogeriatric Society.

[ref-66] Reynolds SL, Silverstein M (2003). Observing the onset of disability in older adults. Social Science & Medicine.

[ref-67] Roberts HC, Denison HJ, Martin HJ, Patel HP, Syddall H, Cooper C, Sayer AA (2011). A review of the measurement of grip strength in clinical and epidemiological studies: Towards a standardised approach. Age and Ageing.

[ref-68] Rockwood K, Hogan DB, MacKnight C (2000). Conceptualisation and measurement of frailty in elderly people. Drugs & Aging.

[ref-69] Rouch I, Dorey J-M, Strippoli M-PF, Gholam M, Marques-Vidal P, Laurent B, von Gunten A, Preisig M (2021). Does cognitive functioning predict chronic pain in older adult? Results from the CoLaus—PsyCoLaus longitudinal study. The Journal of Pain.

[ref-70] Schutter N, Holwerda TJ, Comijs HC, Stek ML, Peen J, Dekker JJM (2022). Loneliness, social network size and mortality in older adults: A meta-analysis. European Journal of Ageing.

[ref-71] Sobhani A, Fadayevatan R, Sharifi F, Kamrani AA, Ejtahed H-S, Hosseini RS, Mohamadi S, Fadayevatan A, Mortazavi S (2021). The conceptual and practical definitions of frailty in older adults: a systematic review. Journal of Diabetes and Metabolic Disorders.

[ref-72] Sol K, Sharifian N, Manly JJ, Brickman AM, Zahodne LB (2021). Associations between loneliness, reading ability and episodic memory in non-hispanic black and white older adults. Archives of Clinical Neuropsychology.

[ref-73] Sörman DE, Ljungberg JK, Rönnlund M (2018). Reading habits among older adults in relation to level and 15-year changes in verbal fluency and episodic recall. Frontiers in Psychology.

[ref-74] Sousa-Santos AR, Amaral (2017) TF (2017). Differences in handgrip strength protocols to identify sarcopenia and frailty—A systematic review. BMC Geriatrics.

[ref-75] Spexoto MCB, Ramírez PC, de Oliveira Máximo R, Steptoe A, de Oliveira C, Alexandre T da S (2022). European Working Group on Sarcopenia in Older People 2010 (EWGSOP1) and 2019 (EWGSOP2) criteria or slowness: Which is the best predictor of mortality risk in older adults?. Age and Ageing.

[ref-76] Stuck AE, Siu AL, Wieland GD, Adams J, Rubenstein LZ (1993). Comprehensive geriatric assessment: a meta-analysis of controlled trials. Lancet.

[ref-77] Su S, Shi L, Zheng Y, Sun Y, Huang X, Zhang A, Que J, Sun X, Shi J, Bao Y, Deng J, Lu L (2022). Leisure activities and the risk of dementia: a systematic review and meta-analysis. Neurology.

[ref-78] Sun R, Ge B, Wu S, Li H, Lin L (2023). Optimal cut-off MoCA score for screening for mild cognitive impairment in elderly individuals in China: a systematic review and meta-analysis. Asian Journal of Psychiatry.

[ref-79] Tangen GG, Langballe EM, Strand BH (2020). Subjective memory impairment, instrumental activities of daily living and longitudinal effect on mortality among older adults in a population-based cohort study: The HUNT Study. Scandinavian Journal of Public Health.

[ref-80] Vass M, Hendriksen C (2016). In Denmark, there is a lack of consensus in the definition of frailty. Ugeskrift for Laeger.

[ref-81] Verghese J, Lipton RB, Katz MJ, Hall CB, Derby CA, Kuslansky G, Ambrose AF, Sliwinski M, Buschke H (2003). Leisure activities and the risk of dementia in the elderly. The New England Journal of Medicine.

[ref-82] Vermeiren S, Vella-Azzopardi R, Beckwée D, Habbig AK, Scafoglieri A, Jansen B, Bautmans I, Bautmans I, Verté D, Beyer I, Petrovic M, Donder LD, Kardol T, Rossi G, Clarys P, Scafoglieri A, Cattrysse E, Hert P de, Jansen B (2016). Frailty and the prediction of negative health outcomes: a meta-analysis. Journal of the American Medical Directors Association.

[ref-83] Wang DXM, Yao J, Zirek Y, Reijnierse EM, Maier AB (2020). Muscle mass, strength, and physical performance predicting activities of daily living: A meta-analysis. Journal of Cachexia, Sarcopenia and Muscle.

[ref-84] World Health Organization (2001). International classification of functioning, disability and health, ICF.

[ref-85] Wu F, Sheng Y (2019). Social support network, social support, self-efficacy, health-promoting behavior and healthy aging among older adults: A pathway analysis. Archives of Gerontology and Geriatrics.

[ref-86] Zimmer Z, Fraser K, Grol-Prokopczyk H, Zajacova A (2022). A global study of pain prevalence across 52 countries: examining the role of country-level contextual factors. Pain.

