# Supplementary material for: Development and content validity of the evaluation of multidimensional functioning and risks in aging scale
Source: PeerJ. 2025 Dec 9;13:e20108. doi: 10.7717/peerj.20108 (PMC12700117; doi:10.7717/peerj.20108)
Supplement: Supplemental Information 1 [file peerj-13-20108-s001.docx]

**EMFRA first preliminary version (EMFRA-P1) after review and synthesis of the literature.**

| **Item** | **Question / Task** | **Measurement procedure** | **Test result** | **Response options** | | **Score** |
| --- | --- | --- | --- | --- | --- | --- |
| ***Physical functioning*** | | | | | |  |
| Item nº1. Hand-grip strength | *Test de fuerza de prensión manual.* | El paciente debe estar sentado, con los brazos a lo largo del tronco, codo flexionado a 90º en pronosupinación neutra sin apoyar el antebrazo. Se realizarán 3 intentos con su mano dominante (o menos afecta), empleando un dinamómetro de prensión manual. Cada contracción debe durar entre 3-6 segundos, descansando 1 minuto entre intentos. No utilice comandos verbales para animar a alcanzar la fuerza máxima durante la medición. Se tomará el valor más alto obtenido.  Si utiliza un dinamómetro Jamar o similar, emplee el agarre ajustado a la 2ª muesca más corta. Si utiliza un dinamómetro con agarre ajustable, elija la distancia más cómoda para el paciente. | 1ª intento (kg) =  2ª intento (kg) =  3ª intento (kg) = | Male: | - <29 kg - ≥29 kg - ≥36 kg | 0 puntos  1 punto  2 puntos |
|  |  |  | 1ª intento (kg) =  2ª intento (kg) =  3ª intento (kg) = | Female: | - <18 kg - ≥18 kg - ≥23 kg | 0 puntos  1 punto  2 puntos |
| Item nº2. Gait speed | *Test de velocidad normal de marcha lineal al recorrer 2,4 m.* | El paciente debe caminar una distancia de 2,4 m en línea recta a velocidad normal. Se ampliará el recorrido añadiendo 1 m extra al principio y otro metro extra al final del recorrido. El paciente caminará desde el metro inicial, recorrerá los 2,4 m y continuará caminando hasta sobrepasar el metro final. Cronometre el tiempo durante los 2,4 m.  Si utiliza dispositivos de asistencia a la marcha, debe usarlo en el test. Se realizarán 2 intentos, descansando 30-90 segundos entre intentos. Se tomará el menor tiempo obtenido. | 1ª intento (m/s) =  2ª intento (m/s) = | - Incapaz de caminar - >0.8 m/s - ≤0.8 m/s | | 0 puntos  1 punto  2 puntos |
| Item nº3. Gait assistance | *¿Suele necesitar la ayuda de alguien o de algún dispositivo (bastón, muletas, andador, etc.) para caminar?* | HC | - | - Frecuentemente - En ocasiones - Casi nunca | | 0 puntos  1 punto  2 puntos |
| Item nº4. Balance | *¿Suele perder el equilibrio al realizar actividades en el día a día?* | HC | - | - Frecuentemente - En ocasiones - Casi nunca | | 0 puntos  1 punto  2 puntos |
| Item nº5. Sedentarism | *¿Cuántas horas durante el día pasa sentado, recostado o tumbado?*  Indique el número de horas: _____ | HC | h = | - ≥11 h - >9 h - ≤9 h | | 0 puntos  1 punto  2 puntos |
| Item nº6. Fatigue | *¿Suele sentirse fatigado o cansado?* | HC | - | - Frecuentemente - En ocasiones - Casi nunca | | 0 puntos  1 punto  2 puntos |
| ***Cognitive functioning*** | | | | | |  |
| Item nº7. Language | *Indique 5 palabras que rimen con la palabra “risa”. No repita la misma palabra varias veces. Puede utilizar rimas asonantes como consonantes.*  Por ejemplo: La palabra “taza” rima de forma consonante con “terraza” y asonante con “capa”.  Anote las palabras que el participante ha mencionado: | Se enuncia la frase indicada en cursiva. Se le mencionará un ejemplo de rima consonante y de rima asonante ["Por ejemplo, la palabra “taza” rima de forma consonante con “terraza” y asonante con “capa”].  El evaluador debe anotar las palabras mencionadas por el participante y analizar si el resultado del test ha sido correcto o incorrecto.  El participante no lee el documento, el test es completamente oral. | - Correcto: Indica un total de 5 palabras, que riman y no se repiten - Incorrecto: Indica menos de 5 palabras, alguna no rima o alguna se repite, o es incapaz de realizar el test | - 0 tests correctos - 1 test correcto - 2 tests correctos | | 0 puntos  1 punto  2 puntos |
|  | Indique el número total de vocales “a” que contiene la siguiente frase: “La patata es una hortaliza nutritiva” | Se enuncia al participante la información previamente indicada. El participante no lee el documento, el test es completamente oral. | - Correcto: 8 letras “a” - Incorrecto: ≠8 letras “a”, o es incapaz de realizar el test |  |  |  |
| Item nº8. Arithmetic for money management | *¿Cuántas monedas de 50 céntimos se necesitan para alcanzar 6 euros?* | El paciente dispone de 1 minuto para completar el test. Durante este tiempo, el paciente puede dar hasta 2 respuestas. Después de cada respuesta, se le preguntará: “¿Esa es su respuesta final?”.  No debe mencionarle al paciente que cuenta únicamente con 2 oportunidades para responder, ni el tiempo máximo del que dispone. | - Correcto: 12 - Incorrecto: ≠12, da 3 o más respuestas, tarda más de 1 minuto, o incapaz de realizar el test | - 0 tests correctos - 1 test correcto - 2 tests correctos | | 0 puntos  1 punto  2 puntos |
|  | *Si un artículo cuesta 11 euros con 50 céntimos y usted paga con un billete de 20 euros ¿Cuánto cambio recibiría?* | El paciente dispone de 1 minuto para completar el test. Durante este tiempo, el paciente puede dar hasta 2 respuestas. Después de cada respuesta, se le preguntará: “¿Esa es su respuesta final?”.  No debe mencionarle al paciente que cuenta únicamente con 2 oportunidades para responder, ni el tiempo máximo del que dispone. | - Correcto: 8,50 euros - Incorrecto: ≠8,50 euros, da 3 o más respuestas, tarda más de 1 minuto, o incapaz de realizar el test |  |  |  |
| Item nº9. Attention-inhibition | *A continuación, voy a recitar 6 números. Quiero que dé un golpecito con la mano cada vez que uno de los números mencionados contenga el digito “2”. Si el número mencionado no contiene el digito “2”, no dé el golpecito.*  *Por ejemplo, si digo el número “32”, debes dar el golpecito, pero si menciono el número “15” no debes dar el golpecito. Vamos a practicar con 3 números de ejemplo. ¿Está listo?:*  *32; 15; 23*  *Ahora, haremos la prueba final. Esta vez con 6 números. Solo dé el golpecito si uno de los números mencionados contiene el dígito “2”. Si no contiene el dígito “2” no dé el golpe. ¿Está listo?:*  *18; 12; 25; 31; 42; 30.* | En esta prueba debe recitar una serie de números, enunciándolos a un ritmo constante de 20 bpm utilizando un metrónomo. No debe dar comentarios al paciente sobre cómo ha realizado la prueba de ejemplo. Marque con una cruz aquellos números en los que golpee el paciente. | **Atención:**   1. Golpea en todos los números “12”, “25” y “42” 2. Golpea en dos de los números “12”, “25” y “42” 3. Golpea en uno o ninguno de los números “12”, “25” y “42”, o es incapaz de realizar el test   **Inhibición:**   1. No golpea en ninguno de los números “18”, ni “31”, ni “30”. 2. Golpea en uno de los números “18”, “31”, o “30”.   Golpea en dos o en todos los números “18”, “31”, y/o “30”, o es incapaz de realizar el test | - 2 opciones B, o 1 opción C - 1 opción A y 1 opción B - 2 opciones A | | 0 puntos  1 punto  2 puntos |
| Item nº10. Memory | *¿Tiene dificultad para recordar eventos recientes, o tareas diarias?* | HC | - | - Mucha dificultad - Algo de dificultad - Muy poca dificultad | | 0 puntos  1 punto  2 puntos |
| Item nº11. Concentration | *¿Tiene dificultad para concentrarse durante largos periodos de tiempo?* | HC | - | - Mucha dificultad - Algo de dificultad - Muy poca dificultad | | 0 puntos  1 punto  2 puntos |
| Item nº12. Cognitive activities | *¿Suele realizar actividades cognitivas como leer libros, revistas o periódicos, escribir, hacer crucigramas, sopas de letras, puzles, sudokus, jugar a juegos de mesa o de cartas, participar en debates grupales organizados o tocar instrumentos musicales?* | H**C** | - | - Casi nunca - En ocasiones - Frecuentemente | | 0 puntos  1 punto  2 puntos |
| ***Emotional status*** | | | | | |  |
| Item nº13. Loneliness | *¿Suele sentirse solo?* | HC | - | - Frecuentemente - En ocasiones - Casi nunca | | 0 puntos  1 punto  2 puntos |
| Item nº14. Sadness | *¿Suele sentirse triste?* | HC | - | - Frecuentemente - En ocasiones - Casi nunca | | 0 puntos  1 punto  2 puntos |
| Item nº15. Fear | *¿Suele sentir que algo malo le puede suceder?* | HC | - | - Frecuentemente - En ocasiones - Casi nunca | | 0 puntos  1 punto  2 puntos |
| Item nº16. Irritability | *¿Suele irritarse o enfadarse con facilidad?* | HC | - | - Frecuentemente - En ocasiones - Casi nunca | | 0 puntos  1 punto  2 puntos |
| Item nº17. Self-efficacy | *¿Suele tener dificultad para hacer frente a situaciones estresantes?* | HC | - | - Frecuentemente - En ocasiones - Casi nunca | | 0 puntos  1 punto  2 puntos |
| Item nº18. Life satisfaction | *¿Cómo describiría su nivel de satisfacción con la vida?* | HC | - | - Muy poco satisfecho - Algo satisfecho - Muy satisfecho | | 0 puntos  1 punto  2 puntos |
| ***Social status*** | | | |  |  |  |
| Item nº19. Economical support | *¿Es capaz de satisfacer sus necesidades básicas (alimentación adecuada, alojamiento seguro y cómodo, atención médica, higiene personal, vestimenta adecuada, seguridad y protección) con el apoyo económico del que dispone?* | HC | - | - Con gran dificultad - Con cierta dificultad - Sin dificultad | | 0 puntos  1 punto  2 puntos |
| Item nº20. Family interaction | *¿Suele pasar tiempo con sus familiares cercanos?* | HC | - | - Casi nunca - En ocasiones - Frecuentemente | | 0 puntos  1 punto  2 puntos |
| Item nº21. Social interaction | *¿Suele pasar tiempo con amigos o conocidos?* | HC | - | - Casi nunca - En ocasiones - Frecuentemente | | 0 puntos  1 punto  2 puntos |
| Item nº22. Participation in ludic and social activities | *¿Suele participar en actividades lúdicas o de ocio con otras personas?* | HC | - | - Casi nunca - En ocasiones - Frecuentemente | | 0 puntos  1 punto  2 puntos |
| Item nº23. Communication ability | *¿Tiene dificultad para comunicarse por teléfono u otros dispositivos?* | HC | - | - Mucha dificultad - Algo de dificultad - Muy poca dificultad | | 0 puntos  1 punto  2 puntos |
| Item nº24. Receiving help | *¿Puede contar con la ayuda de otra persona en caso de necesitarla?* | HC | - | - Casi nunca - En ocasiones - Frecuentemente | | 0 puntos  1 punto  2 puntos |
