# Supplementary material for: Development and content validity of the evaluation of multidimensional functioning and risks in aging scale
Source: PeerJ. 2025 Dec 9;13:e20108. doi: 10.7717/peerj.20108 (PMC12700117; doi:10.7717/peerj.20108)
Supplement: Supplemental Information 2 [file peerj-13-20108-s002.docx]

**EMFRA second preliminary version (EMFRA-P2) with highlighted changes after content validity by experts.**

| **Evaluación Multidimensional de Funcionamiento y Riesgos en el Envejecimiento (EMFRA)** | | | | | | | | |
| --- | --- | --- | --- | --- | --- | --- | --- | --- |
| **Ítem 1. Test de fuerza.**  El paciente debe estar sentado, con los brazos a lo largo del tronco, codo flexionado a 90º en pronosupinación neutra sin apoyar el antebrazo. Se realizarán 3 intentos con su mano dominante (o menos afecta), empleando un dinamómetro de prensión manual. Cada contracción debe durar entre 3-6 segundos, descansando 1 minuto entre intentos. ~~No~~ Utilice comandos verbales para animar a alcanzar la fuerza máxima durante el intento. ~~Se tomará el valor más alto obtenido.~~  Si utiliza un dinamómetro Jamar o similar, emplee el agarre ajustado a la 2ª muesca más corta. Si utiliza un dinamómetro con agarre ajustable, elija la distancia más cómoda para el paciente.  Anote los kg alcanzados en cada intento:  1ª Intento: ________ 2ª Intento: ________ 3ª Intento: ________  Básese en el valor más alto obtenido para calificar la puntuación. | | | | **Hombre** | - ≥36 kg | - ≥29 kg | | - <29 kg |
|  |  |  |  | **Mujer** | - ≥23 kg | - ≥18 kg | | - <18 kg |
| **Ítem 2. Test de marcha.**  El paciente debe caminar una distancia de 2,4 m en línea recta a velocidad normal. Se ampliará el recorrido añadiendo 1 m extra al principio y otro metro extra al final del recorrido. El paciente caminará desde el metro inicial, recorrerá los 2,4 m y continuará caminando hasta sobrepasar el metro final. Cronometre el tiempo durante los 2,4 m.  Si utiliza dispositivos de asistencia a la marcha debe usarlo en el test. Se realizarán 2 intentos con1 minuto de descanso entre ellos. ~~Se tomará el menor tiempo obtenido.~~  Anote el tiempo (segundos) alcanzados en cada intento:  1ª Intento: ________ 2ª Intento: ________  Básese en el valor más alto obtenido para calificar la puntuación. | | | | | - ≤3 s | - >3 s | | - Incapaz de caminar |
| **Ítem 3.** *¿Suele necesitar la ayuda de alguien o de algún dispositivo (bastón, muletas, andador, etc.) para caminar?* | | | | | - Casi nunca | - En ocasiones | | - Frecuentemente |
| **Ítem 4.** *¿Suele perder el equilibrio al realizar actividades en el día a día?* | | | | | - Casi nunca | - En ocasiones | | - Frecuentemente |
| **Ítem 5.** *¿Cuántas horas durante el día pasa sentado, recostado o tumbado?*  Indique el número de horas: ________ | | | | | - ≤9 horas | - >9 horas | | - ≥11 horas |
| **Ítem 6.** *¿Suele sentirse fatigado o cansado?* | | | | | - Casi nunca | - En ocasiones | | - Frecuentemente |
| **Ítem 7. Tests de cálculo.**  El paciente dispone de 1 minuto para completar cada uno de los siguientes tests. Durante este tiempo, el paciente puede dar hasta 2 respuestas. Después de cada respuesta, se le preguntará: *¿Esa es su respuesta final?*  No debe mencionarle al paciente que cuenta únicamente con 2 oportunidades para responder, ni el tiempo máximo del que dispone.  Los cálculos deben realizarse mentalmente. El paciente puede utilizar sus manos como apoyo, pero no se permite el uso de papel ni calculadora. | | | | | - 2 test correctos | - 1 test correcto | | - 0 test correctos |
| *¿Cuántas monedas de 50 céntimos se necesitan para alcanzar 6 euros?* | - Correcto: 12 - Incorrecto: ≠12, da 3 o más respuestas, tarda más de 1 minuto, o incapaz de realizar el test | | | |  |  |  |  |
| *Si un artículo cuesta 11 euros con 50 céntimos y usted paga con un billete de 20 euros ¿Cuánto cambio recibiría?* | | - Correcto: 8,50 euros - Incorrecto: ≠8,50 euros, da 3 o más respuestas, tarda más de 1 minuto, o incapaz de realizar el test | | |  |  |  |  |
| **Ítem 8. Test de Atención e inhibición*.***  En esta prueba debe recitar una serie de números, enunciándolos a un ritmo constante de 20 bpm utilizando un metrónomo. Puede seguir este ritmo utilizando un cronómetro. No debe dar comentarios al paciente sobre cómo ha realizado la prueba de ejemplo. Marque con una cruz aquellos números en los que golpee el paciente. | | | | | - 2 opciones A | - 1 opción A y 1 opción B | | - 2 opciones B o 1 opción C |
| *A continuación, voy a recitar 6 números. Quiero que dé un golpecito con la mano cada vez que uno de los números mencionados contenga el digito “2”. Si el número mencionado no contiene el digito “2”, no dé el golpecito.*  *Por ejemplo, si digo el número “32”, debes dar el golpecito, pero si menciono el número “15” no debes dar el golpecito. Vamos a practicar con 3 números de ejemplo. ¿Está listo?:*  *32; 15; 23*  *Ahora, haremos la prueba final. Esta vez con 6 números. Solo dé el golpecito si uno de los números mencionados contiene el dígito “2”. Si no contiene el dígito “2” no dé el golpe. ¿Está listo?:*   \| □ \| □ \| □ \| □ \| □ \| □ \| \| --- \| --- \| --- \| --- \| --- \| --- \| \| 18 \| 12 \| 25 \| 31 \| 42 \| 30 \| | | | **Atención:**   1. Golpea en todos los números “12”, “25” y “42” 2. Golpea solamente en dos de los números “12”, “25” o “42” 3. Golpea en uno o ninguno de los números “12”, “25” o “42”, o es incapaz de realizar el test | |  |  |  |  |
|  |  |  | **Inhibición:**   1. No golpea en ninguno de los números “18” ni “31” ni “30” 2. Golpea en uno de los números “18”, “31” o “30” 3. Golpea en dos o en todos los números “18”, “31” y/o “30”, o es incapaz de realizar el test | |  |  |  |  |
| **Ítem 9.** *¿Tiene dificultad para recordar eventos recientes, o tareas diarias?* | | | | | - Casi nunca | - En ocasiones | | - Frecuentemente |
| **Ítem 10.** *¿Tiene dificultad para concentrarse durante largos periodos de tiempo?* | | | | | - Casi nunca | - En ocasiones | | - Frecuentemente |
| **Ítem 11.** *¿Suele realizar actividades cognitivas como leer libros, revistas o periódicos, escribir, hacer crucigramas, sopas de letras, puzles, sudokus, jugar a juegos de mesa o de cartas, participar en debates grupales organizados o tocar instrumentos musicales?* | | | | | - Casi nunca | - En ocasiones | | - Frecuentemente |
| **Ítem 12.** *¿Suele sentirse solo?* | | | | | - Casi nunca | - En ocasiones | | - Frecuentemente |
| **Ítem 13.** *¿Suele sentirse triste?* | | | | | - Casi nunca | - En ocasiones | | - Frecuentemente |
| **Ítem 14.** *¿Suele irritarse o enfadarse con facilidad?* | | | | | - Casi nunca | - En ocasiones | | - Frecuentemente |
| **Ítem 15.** *¿Suele tener dificultad para hacer frente a situaciones estresantes?* | | | | | - Casi nunca | - En ocasiones | | - Frecuentemente |
| **Ítem 16.** *¿Cómo describiría su nivel de satisfacción con la vida actualmente?* | | | | | - Muy poco satisfecho | - Algo satisfecho | | - Muy satisfecho |
| **Ítem 17.** *¿Es capaz de satisfacer sus necesidades básicas (alimentación adecuada, alojamiento seguro y cómodo, atención médica, higiene personal, vestimenta adecuada, seguridad y protección) con el apoyo económico del que dispone?* | | | | | - ~~Con gran dificultad~~ Muy difícil | - ~~Con cierta dificultad~~Algo difícil | | - ~~Sin dificultad~~ Nada difícil |
| **Ítem 18.** *¿Suele pasar tiempo con sus familiares ~~cercanos~~?* | | | | | - Casi nunca | - En ocasiones | | - Frecuentemente |
| **Ítem 19.** *¿Suele pasar tiempo con amigos o conocidos?* | | | | | - Casi nunca | - En ocasiones | | - Frecuentemente |
| **Ítem 20.** *¿Suele participar en actividades lúdicas o de ocio con otras personas?* | | | | | - Casi nunca | - En ocasiones | | - Frecuentemente |
| **Ítem 21.** *¿Tiene dificultad para comunicarse por teléfono u otros dispositivos?* | | | | | - Casi nunca | - En ocasiones | | - Frecuentemente |
| **Ítem 22.** *¿Puede contar con la ayuda de otra persona en caso de necesitarla?* | | | | | - Casi nunca | - En ocasiones | | - Frecuentemente |
| **Sistema de puntuación.**  La puntuación total de la escala varía de 0 a 44 puntos. Los ítems se puntúan con 2, 1 o 0 puntos, para la 1ª, 2ª, y 3ª opción de respuesta (situadas de izquierda a derecha). Todos los ítems siguen el formato de puntuación anterior, excepto los ítems nº11, 16, 18-20, y 22, donde se puntúa con 0, 1, y 2 puntos respectivamente para la 1ª, 2ª, y 3ª opción de respuesta (situadas de izquierda a derecha).  Para calcular la puntuación total sume las puntuaciones de todos los ítems. | | | | | | | **Puntuación total:**  ________ / 44 | |
