# Supplementary material for: Development and content validity of the evaluation of multidimensional functioning and risks in aging scale
Source: PeerJ. 2025 Dec 9;13:e20108. doi: 10.7717/peerj.20108 (PMC12700117; doi:10.7717/peerj.20108)
Supplement: Supplemental Information 3 [file peerj-13-20108-s003.docx]

| **Evaluation of Multidimensional Functioning and Risks in Aging (EMFRA)** | | | | | | | |
| --- | --- | --- | --- | --- | --- | --- | --- |
| **Item 1. Strength Test.**  **Instructions: The patient must be seated with arms alongside the trunk, the elbow flexed at 90° in a neutral pronosupination position without supporting the forearm. Three measurements will be taken using the dominant hand (or the least affected hand) with a handgrip dynamometer. Each contraction should last between 3 to 6 seconds, with a 1-minute rest between measurements. Provide verbal encouragement to achieve maximal strength during each attempt.**  **If using a Jamar dynamometer, set the grip to the second shortest notch. If using an adjustable-grip dynamometer, select the most comfortable grip distance for the patient.**  **Record the kilograms obtained in each measurement:**  **1st Trial: _____ 2nd Trial: _____ 3rd Trial: _____**  **The highest value obtained should be used for scoring.** | | | **Men** | - **≥36 kg** | - **≥29 kg** | | - **<29 kg** |
|  |  |  | **Women** | - **≥23 kg** | - **≥18 kg** | | - **<18 kg** |
| **Item 2. Gait Test.**  **Instructions: The patient must walk a straight-line distance of 2.4 meters at a normal speed. The path will be extended by adding 1 extra meter at the beginning and another at the end. The patient should start walking from the initial meter, complete the 2.4-meter path, and continue walking past the final meter. Time should be recorded for the 2.4-meter segment.**  **If the patient uses assistive walking devices such as a cane, walker, or crutches, they should use them during the test. Two measurements will be taken, with a 1-minute rest between them.**  **Record the time (seconds) for each measurement:**  **1st Trial: ______________ 2nd Trial: ______________**  **The lowest recorded value should be used for scoring.** | | | | - **≤3 s** | - **>3 s** | | - **Unable to walk** |
| **Item 3. *“Do you usually need assistance from someone or a device to walk? For example, a cane, crutches, walker, or others.”*** | | | | - **Never** | - **Sometimes** | | - **Frequently** |
| **Item 4. *“Do you usually lose your balance while performing daily activities?”*** | | | | - **Never** | - **Sometimes** | | - **Frequently** |
| **Item 5. *“Do you usually feel fatigued or tired?”*** | | | | - **Never** | - **Sometimes** | | - **Frequently** |
| **Item 6. Calculation Tests.**  **Instructions: The patient has 1 minute to complete each of the following tests. Within this period, the patient can provide up to 2 responses. After each response, ask: *“Is that your final answer?”***  **Do not inform the patient that they are limited to 2 attempts or the total time available.**  **The calculations must be performed mentally. The patient may use their hands for support but cannot use paper or a calculator.** | | | | - **2 correct tests** | - **1 correct test** | | - **0 correct tests** |
| **Test nº1: *“How many 50-cent coins are needed to make up 6 euros?”*** | - **Correct: 12** - **Incorrect: ≠12, provides 3 or more responses, exceeds 1-minute limit, or unable to perform the test.** | | |  |  |  |  |
| **Test nº2: *“If a product costs 11 euros and 50 cents, and you pay with a 20-euro bill, how much change will you receive?”*** | - **Correct: 8,50** - **Incorrect: ≠8.50, provides 3 or more responses, exceeds 1-minute limit, or unable to perform the test.** | | |  |  |  |  |
| **Item 7. Attention and Inhibition Test.**  **Instructions: In this test, recite a series of numbers at a constant rate of 1 number every 3 seconds using a stopwatch. Do not provide feedback on the example trial. Mark with a cross the numbers where the patient tapped to respond.** | | | | - **2 options A** | - **1 option A and 1 option B** | | - **2 options B or 1 option C** |
| ***“I will now read a series of numbers sequentially. I want you to tap your hand whenever you hear a number that contains the digit '2'. If the number does not contain a '2', do not tap.***  ***For example, if I say '32', you should tap. If I say '15', you should not tap.”***  ***“We will practice with 3 example numbers. Are you ready?”:***  ***32; 15; 23***  ***“Now, we will conduct the final test with 6 numbers. Tap only if the number contains the digit '2'. If it does not, do not tap. Are you ready?”:***   \| **□** \| **□** \| **□** \| **□** \| **□** \| **□** \| \| --- \| --- \| --- \| --- \| --- \| --- \| \| **18** \| **12** \| **25** \| **31** \| **42** \| **30** \|   **-** | | **For numbers 12, 25, and 42. Select one option:**   1. **Taps 3 numbers.** 2. **Taps 2 numbers.** 3. **Taps 1 number, does not tap any, or unable to complete the test.** | |  |  |  |  |
|  |  | **For numbers 18, 31, and 30. Select one option:**   1. **Does not tap any.** 2. **Taps 1 number.** 3. **Taps 2 or 3 numbers, or unable to complete the test.** | |  |  |  |  |
| **Item 8. *“Do you have difficulty remembering recent events?”*** | | | | - **Never** | - **Sometimes** | | - **Frequently** |
| **Item 9. *“Do you have difficulty concentrating for long periods?”*** | | | | - **Never** | - **Sometimes** | | - **Frequently** |
| **Item 10.**  **Instructions: Select the response based on the most frequently performed activity.**  ***“Do you frequently engage in activities such as reading, writing, solving crosswords, word searches, puzzles, Sudoku, playing board or card games, participating in discussions, or playing musical instruments?”*** | | | | - **Never** | - **Sometimes** | | - **Frequently** |
| **Item 11. *“Do you usually feel lonely?”*** | | | | - **Never** | - **Sometimes** | | - **Frequently** |
| **Item 12. “*Do you usually feel sad?”*** | | | | - **Never** | - **Sometimes** | | - **Frequently** |
| **Item 13. *“Do you usually get irritated or angry with ease?”*** | | | | - **Never** | - **Sometimes** | | - **Frequently** |
| **Item 14. *“Do you have difficulty coping with challenging situations?”*** | | | | - **Never** | - **Sometimes** | | - **Frequently** |
| **Item 15. “*Are you currently satisfied with your life?”*** | | | | - **Not satisfied at all** | - **Somewhat satisfied** | | - **Very satisfied** |
| **Item 16. *“Given your current financial situation, do you have difficulty covering basic needs such as food, housing, medical care, hygiene, and clothing?”*** | | | | - **Never** | - **Sometimes** | | - **Frequently** |
| **Item 17. “*Do you usually spend time with family members?”*** | | | | - **Never** | - **Sometimes** | | - **Frequently** |
| **Item 18. *“Do you usually spend time with friends or acquaintances?”*** | | | | - **Never** | - **Sometimes** | | - **Frequently** |
| **Item 19. *“Do you usually participate in social or recreational activities with others? For example, meeting friends, dancing, going to the cinema or theater.”*** | | | | - **Never** | - **Sometimes** | | - **Frequently** |
| **Item 20. *“Do you have difficulty communicating via telephone or other devices?”*** | | | | - **Never** | - **Sometimes** | | - **Frequently** |
| **Item 21. *“If you need help, do you have someone to rely on?”*** | | | | - **Never** | - **Sometimes** | | - **Frequently** |
| **Scoring System.**  **The total scale score ranges from 0 to 42 points. Items are scored with 2, 1, or 0 points for the first, second, and third response options (left to right). All items follow this scoring format except for items 10, 15, 17-19, and 21, where the scoring is 0, 1, and 2 points respectively for the first, second, and third response options (left to right).**  **To calculate the total score, sum the scores of all items.** | | | | | | **Total score:**  **________ / 42** | |
