# Supplementary material for: Development and content validity of the evaluation of multidimensional functioning and risks in aging scale
Source: PeerJ. 2025 Dec 9;13:e20108. doi: 10.7717/peerj.20108 (PMC12700117; doi:10.7717/peerj.20108)
Supplement: Supplemental Information 4 [file peerj-13-20108-s004.docx]

| **Evaluación Multidimensional de Funcionamiento y Riesgos en el Envejecimiento (EMFRA)** | | | | | | | |
| --- | --- | --- | --- | --- | --- | --- | --- |
| **Ítem 1. Test de fuerza.**  **Instrucciones:** El paciente debe estar sentado, con los brazos a lo largo del tronco, codo flexionado a 90º en pronosupinación neutra sin apoyar el antebrazo. Se realizarán 3 mediciones con su mano dominante (o menos afecta), empleando un dinamómetro de prensión manual. Cada contracción debe durar entre 3-6 segundos, descansando 1 minuto entre mediciones. Utilice comandos verbales para animar a alcanzar la fuerza máxima durante el intento.  Si utiliza un dinamómetro Jamar, emplee el agarre ajustado a la 2ª muesca más corta. Si utiliza un dinamómetro con agarre ajustable, elija la distancia más cómoda para el paciente.  Anote los kg alcanzados en cada medición:  1ª Medición: ________ 2ª Medición: ________ 3ª Medición: ________  Básese en el valor más alto obtenido para calificar la puntuación. | | | **Hombre** | - **≥36 kg** | - **≥29 kg** | | - **<29 kg** |
|  |  |  | **Mujer** | - **≥23 kg** | - **≥18 kg** | | - **<18 kg** |
| **Ítem 2. Test de marcha.**  **Instrucciones:** El paciente debe caminar una distancia de 2,4 m en línea recta a velocidad normal. Se ampliará el recorrido añadiendo 1 m extra al principio y otro metro extra al final del recorrido. El paciente caminará desde el metro inicial, recorrerá los 2,4 m y continuará caminando hasta sobrepasar el metro final. Cronometre el tiempo durante los 2,4 m.  Si utiliza dispositivos de asistencia a la marcha como bastón, andador, o muleta, debe usarlo en el test. Se realizarán 2 mediciones con 1 minuto de descanso entre ellas.  Anote el tiempo (segundos) de cada medición:  1ª Medición: ______________ 2ª Medición: ______________  Básese en el valor más bajo obtenido para calificar la puntuación. | | | | - **≤3 s** | - **>3 s** | | - **Incapaz de caminar** |
| **Ítem 3. *“¿Suele necesitar la ayuda de alguien o de algún dispositivo para caminar? Por ejemplo, bastón, muletas, andador u otros.”*** | | | | - **Nunca** | - **Algunas veces** | | - **Frecuentemente** |
| **Ítem 4. *“¿Suele perder el equilibrio al realizar actividades en el día a día?”*** | | | | - **Nunca** | - **Algunas veces** | | - **Frecuentemente** |
| **Ítem 5. *“¿Suele sentirse fatigado/a o cansado/a?”*** | | | | - **Nunca** | - **Algunas veces** | | - **Frecuentemente** |
| **Ítem 6. Tests de cálculo.**  **Instrucciones: El paciente dispone de 1 minuto para completar cada uno de los siguientes tests. Durante este tiempo, el paciente puede dar hasta 2 respuestas. Después de cada respuesta, se le preguntará: *“¿Esa es su respuesta final?”***  **No debe mencionarle al paciente que cuenta únicamente con 2 oportunidades para responder, ni el tiempo máximo del que dispone.**  **Los cálculos deben realizarse mentalmente. El paciente puede utilizar sus manos como apoyo, pero no se permite el uso de papel ni calculadora.** | | | | - **2 test correctos** | - **1 test correcto** | | - **0 tests correctos** |
| **Test nº1: “*¿Cuántas monedas de 50 céntimos se necesitan para alcanzar 6 euros?”*** | - **Correcto: 12** - **Incorrecto: ≠12, da 3 o más respuestas, tarda más de 1 minuto, o incapaz de realizar el test** | | |  |  |  |  |
| **Test nº2: “*Si un producto cuesta 11 euros con 50 céntimos y usted paga con un billete de 20 euros, ¿Cuánto cambio recibirá?”*** | - **Correcto: 8,50** - **Incorrecto: ≠8,50, da 3 o más respuestas, tarda más de 1 minuto, o incapaz de realizar el test** | | |  |  |  |  |
| **Ítem 7. Test de Atención e inhibición*.***  **Instrucciones: En esta prueba debe recitar una serie de números, enunciándolos a un ritmo constante de 1 número cada 3 segundos~~,~~ utilizando un cronómetro. No debe dar comentarios al paciente sobre cómo ha realizado la prueba de ejemplo. Marque con una cruz aquellos números en los que golpee el paciente.** | | | | - **2 opciones A** | - **1 opción A y 1 opción B** | | - **2 opciones B o 1 opción C** |
| ***“A continuación, voy a enunciar unos números de forma seguida. Quiero que dé un golpe con la mano cada vez que uno de los números mencionados contenga el dígito “2”. Si el número mencionado no contiene el dígito “2” no dé el golpe.***  ***Por ejemplo, si digo el número “32”, debe dar el golpe, pero si menciono el número “15” no debe dar el golpe.”***  ***“Vamos a practicar con 3 números de ejemplo. ¿Está listo?”:***  ***32; 15; 23***  ***“Ahora, haremos la prueba final. Esta vez con 6 números. Solo debe dar el golpe si uno de los números mencionados contiene el dígito “2”. Si no contiene el dígito “2” no debe dar el golpe. ¿Está listo?”:***   \| **□** \| **□** \| **□** \| **□** \| **□** \| **□** \| \| --- \| --- \| --- \| --- \| --- \| --- \| \| **18** \| **12** \| **25** \| **31** \| **42** \| **30** \|   **-** | | **Para los números 12, 25, y 42. Seleccione una opción:**   1. **Golpea 3 números.** 2. **Golpea 2 números.** 3. **Golpea 1 número, no golpea ninguno, o incapaz de realizar el test.** | |  |  |  |  |
|  |  | **Para los números 18, 31, y 30. Seleccione una opción:**   1. **No golpea ninguno.** 2. **Golpea 1 número.** 3. **Golpea 2 o 3 números, o incapaz de realizar el test.** | |  |  |  |  |
| **Ítem 8. “*¿Tiene dificultad para recordar eventos recientes?”*** | | | | - **Nunca** | - **Algunas veces** | | - **Frecuentemente** |
| **Ítem 9. “*¿Tiene dificultad para concentrarse durante largos periodos de tiempo?”*** | | | | - **Nunca** | - **Algunas veces** | | - **Frecuentemente** |
| **Ítem 10.**  **Instrucciones: Seleccione la respuesta basándose en la actividad que realice con mayor frecuencia.**  ***“¿Suele realizar las siguientes actividades? Por ejemplo, leer, escribir, hacer crucigramas, sopas de letras, puzles, sudokus, jugar a juegos de mesa o de cartas, participar en tertulias o tocar instrumentos musicales.”*** | | | | - **Nunca** | - **Algunas veces** | | - **Frecuentemente** |
| **Ítem 11. “*¿Suele sentirse solo/a?”*** | | | | - **Nunca** | - **Algunas veces** | | - **Frecuentemente** |
| **Ítem 12. “*¿Suele sentirse triste?”*** | | | | - **Nunca** | - **Algunas veces** | | - **Frecuentemente** |
| **Ítem 13. “*¿Suele irritarse o enfadarse con facilidad?”*** | | | | - **Nunca** | - **Algunas veces** | | - **Frecuentemente** |
| **Ítem 14. “*¿Suele tener dificultad para hacer frente a situaciones difíciles?”*** | | | | - **Nunca** | - **Algunas veces** | | - **Frecuentemente** |
| **Ítem 15. “*Actualmente, ¿Está satisfecho/a con su vida?”*** | | | | - **Nada satisfecho** | - **Algo satisfecho** | | - **Muy satisfecho** |
| **Ítem 16. *“Con su situación económica actual, ¿Tiene dificultad para cubrir sus necesidades básicas? Por ejemplo, alimentación, alojamiento, atención médica, higiene y vestimenta.”*** | | | | - **Nunca** | - **Algunas veces** | | - **Frecuentemente** |
| **Ítem 17. “*¿Suele pasar tiempo con sus familiares?”*** | | | | - **Nunca** | - **Algunas veces** | | - **Frecuentemente** |
| **Ítem 18. *“¿Suele pasar tiempo con amigos/as o conocidos/as?”*** | | | | - **Nunca** | - **Algunas veces** | | - **Frecuentemente** |
| **Ítem 19. “*¿Suele participar en actividades lúdicas o de ocio con otras personas? Por ejemplo, quedar con amigos/as, salir a bailar, ir al cine o al teatro.”*** | | | | - **Nunca** | - **Algunas veces** | | - **Frecuentemente** |
| **Ítem 20. “*¿Tiene dificultad para comunicarse por teléfono u otros dispositivos?”*** | | | | - **Nunca** | - **Algunas veces** | | - **Frecuentemente** |
| **Ítem 21. *“En caso de necesitar ayuda, ¿Puede contar con alguien que lo/la ayude?”*** | | | | - **Nunca** | - **Algunas veces** | | - **Frecuentemente** |
| **Sistema de puntuación.**  **La puntuación total de la escala varía de 0 a 42 puntos. Los ítems se puntúan con 2, 1 o 0 puntos, para la 1ª, 2ª, y 3ª opción de respuesta (situadas de izquierda a derecha). Todos los ítems siguen el formato de puntuación anterior, excepto los ítems nº10, 15, 17-19, y 21, donde se puntúa con 0, 1, y 2 puntos respectivamente para la 1ª, 2ª, y 3ª opción de respuesta (situadas de izquierda a derecha).**  **Para calcular la puntuación total sume las puntuaciones de todos los ítems.** | | | | | | **Puntuación total:**  **________ / 42** | |
